# Integrated single-cell analysis of multicellular immune dynamics during hyperacute HIV-1 infection

**DOI:** 10.1038/s41591-020-0799-2

**Published:** 2020-03-23

**Authors:** Samuel W. Kazer, Toby P. Aicher, Daniel M. Muema, Shaina L. Carroll, Jose Ordovas-Montanes, Vincent N. Miao, Ang A. Tu, Carly G. K. Ziegler, Sarah K. Nyquist, Emily B. Wong, Nasreen Ismail, Mary Dong, Amber Moodley, Bonnie Berger, J. Christopher Love, Krista L. Dong, Alasdair Leslie, Zaza M. Ndhlovu, Thumbi Ndung’u, Bruce D. Walker, Alex K. Shalek

**Affiliations:** 1 Ragon Institute of MGH, MIT and Harvard, Cambridge, MA USA; 2 Institute for Medical Engineering and Science (IMES), Massachusetts Institute of Technology, Cambridge, MA USA; 3 Department of Chemistry, Massachusetts Institute of Technology, Cambridge, MA USA; 4 Broad Institute of MIT and Harvard, Cambridge, MA USA; 5 African Health Research Institute, Durban, South Africa; 6 HIV Pathogenesis Programme, Nelson R. Mandela School of Medicine, Doris Duke Medical Research Institute, University of KwaZulu-Natal, Durban, South Africa; 7 Department of Molecular and Cell Biology, University of California, Berkeley, CA USA; 8 Division of Gastroenterology, Boston Children’s Hospital, Boston, MA USA; 9 Harvard Stem Cell Institute, Cambridge, MA USA; 10 Program in Health Sciences and Technology, Harvard Medical School & Massachusetts Institute of Technology, Boston, MA USA; 11 Koch Institute for Integrative Cancer Research, Massachusetts Institute of Technology, Cambridge, MA USA; 12 Department of Chemical Engineering, Massachusetts Institute of Technology, Cambridge, MA USA; 13 Program in Computational and Systems Biology, Massachusetts Institute of Technology, Cambridge, MA USA; 14 Computer Science and Artificial Intelligence Laboratory, Massachusetts Institute of Technology, Cambridge, MA USA; 15 Division of Infectious Diseases, Massachusetts General Hospital, Boston, MA USA; 16 Division of Infection and Immunity, University College London, London, UK; 17 Harvard Medical School, Boston, MA USA; 18 Department of Mathematics, Massachusetts Institute of Technology, Cambridge, MA USA; 19 Howard Hughes Medical Institute, Chevy Chase, MD USA; 20 Max Planck Institute for Infection Biology, Berlin, Germany

**Keywords:** Infectious diseases, Immunology, Genomics, Transcriptomics, Cellular signalling networks

## Abstract

Cellular immunity is critical for controlling intracellular pathogens, but individual cellular dynamics and cell–cell cooperativity in evolving human immune responses remain poorly understood. Single-cell RNA-sequencing (scRNA-seq) represents a powerful tool for dissecting complex multicellular behaviors in health and disease^1,2^ and nominating testable therapeutic targets^3^. Its application to longitudinal samples could afford an opportunity to uncover cellular factors associated with the evolution of disease progression without potentially confounding inter-individual variability^4^. Here, we present an experimental and computational methodology that uses scRNA-seq to characterize dynamic cellular programs and their molecular drivers, and apply it to HIV infection. By performing scRNA-seq on peripheral blood mononuclear cells from four untreated individuals before and longitudinally during acute infection^5^, we were powered within each to discover gene response modules that vary by time and cell subset. Beyond previously unappreciated individual- and cell-type-specific interferon-stimulated gene upregulation, we describe temporally aligned gene expression responses obscured in bulk analyses, including those involved in proinflammatory T cell differentiation, prolonged monocyte major histocompatibility complex II upregulation and persistent natural killer (NK) cell cytolytic killing. We further identify response features arising in the first weeks of infection, for example proliferating natural killer cells, which potentially may associate with future viral control. Overall, our approach provides a unified framework for characterizing multiple dynamic cellular responses and their coordination.

## Abstract (web summary)

Single-cell RNA-sequencing of blood from HIV-1-infected individuals obtained before initiation of antiretroviral therapy provides insights into the initial immune responses during early infection that might shape future outcomes.

## Main

Despite advances in pre-exposure prophylaxis, there were 1.7 million new cases of HIV infection in 2018 (ref. ^6^), highlighting the need for effective HIV vaccines. A better understanding of key immune responses during the earliest stages of infection, especially Fiebig stage I and II, before and at peak viral load, respectively, could help identify future prophylactic and therapeutic targets^7^. Using historical samples, collected before standard-of-care included treatment during acute infection, from the Females Rising through Education, Support and Health (FRESH) study^5^, we assayed evolving immune responses during hyperacute (1–2 weeks post-detection) and acute (3 weeks to 6 months) HIV infection.

**Fig. 1:**
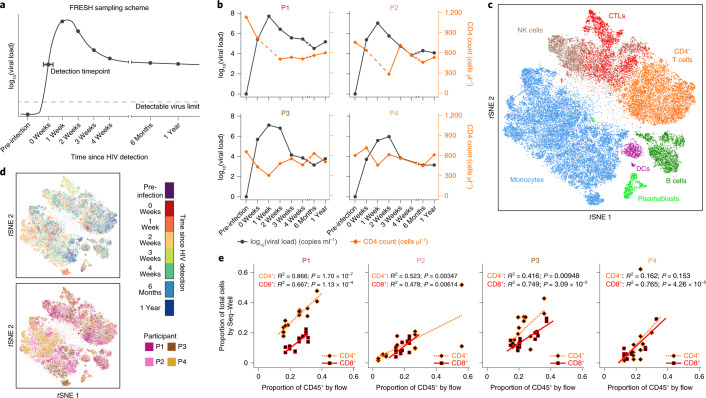
Longitudinal profiling of peripheral immune cells in hyperacute and acute HIV infection by scRNA-seq. **a**, Depiction of the typical trajectory of HIV viral load in the plasma during hyperacute and acute HIV infection adapted from Fiebig et al.^8^ and the timepoints sampled in this study. Since participants were tested twice weekly, there was an uncertainty of up to 3 d in where on the viral load curve the first detectable viremia occurred (error bar is representative). The exact days sampled are available in Supplementary Table 1. **b**, Viral load and CD4^+^ T cell count for four participants assayed in this study. Dotted lines indicate a missing data point for the metric. **c**, *t*-distributed stochastic neighbor embedding (*t*SNE) analysis of PBMCs from all participants and timepoints sampled (*n* = 59,162). Cells are annotated based on differential expression analysis on orthogonally discovered clusters. **d**, *t*SNE in **c** annotated by timepoint (top) and participant (bottom). **e**, Scatter-plot depicting the correlation between cell frequencies of CD4^+^ and CD8^+^ T cells measured by Seq-Well (*n* = 2 array replicates) and FACS (*n* = 1 flow replicate). *R*^2^ values reflect variance described by an F-test for linear regression.

**Extended Data Fig. 1:**
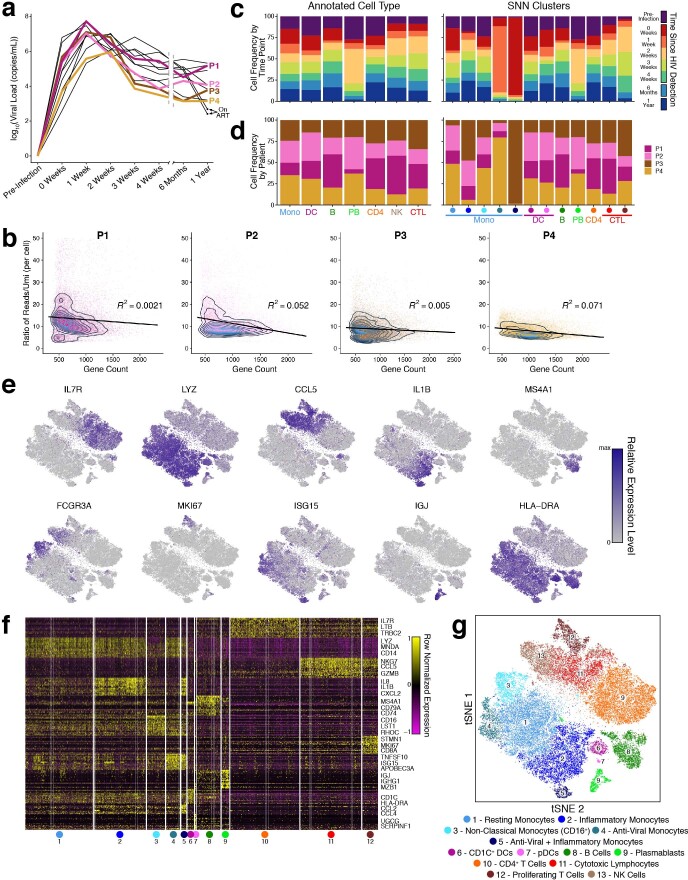
Participant and time point breakdown by cluster and cluster annotation. **a**, Plasma viral load for the 12 participants studied in Ndhlovu et al.^37^ with the four individuals characterized here annotated. **b**, Average ratio of number of reads per number of UMIs measured per single cell. R-squared values reflect variance described by a linear model. Number of cells per participant: P1 – 15,259; P2 – 13,128; P3 – 15,927; P4 – 15,425. **c**, Time point and **d**, participant cell frequency by annotated cell type (left) and shared-nearest neighbors (SNN) clusters (right). **e**, Relative expression levels of exemplary marker genes used for cell type identification projected on the FItSNE. **f**, Heatmap of the top 10 genes differentiating each SNN cluster. Up to 500 random cells are depicted for each cluster. Two-sample Wilcoxon rank sum test; see Supplementary Table 2 for cell numbers. **g**, tSNE embedding of dataset colored by SNN cluster and annotated based on genes in **e, f**, and Supplementary Table 3.

We performed Seq-Well-based massively parallel scRNA-seq on peripheral blood mononuclear cells (PBMCs) from four FRESH participants who became infected with HIV during study. We analyzed multiple timepoints from pre-infection through 1 year following viral detection (Fig. 1a, Supplementary Table 1 and Methods) over which all four demonstrated a rapid rise in plasma viremia and a drop in CD4^+^ T cell counts^8^ (Fig. 1b and Extended Data Fig. 1a). Altogether, we captured 59,162 cells after performing quality controls, with an average of 1,976 cells per participant per timepoint (Extended Data Fig. 1b and Supplementary Table 2).

**Extended Data Fig. 2:**
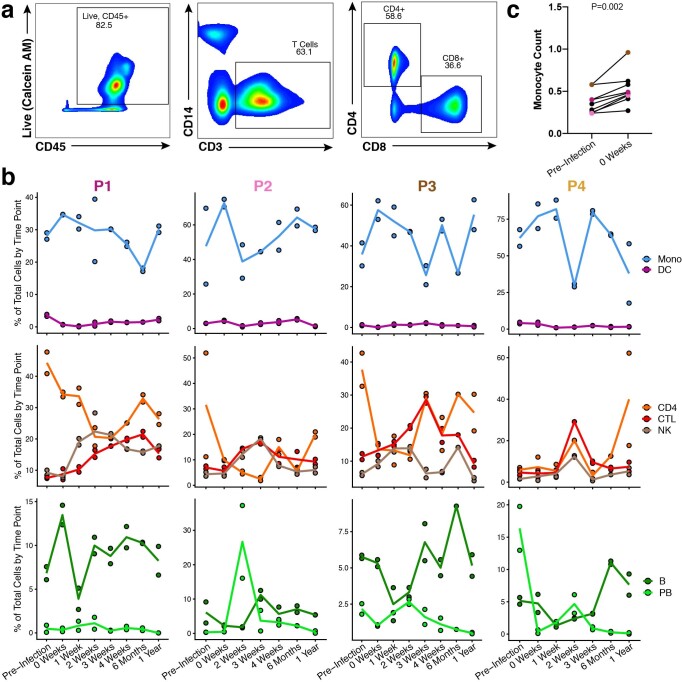
Cell frequency by participant and cell type. **a**, Representative gating scheme for CD4^+^ and CD8^+^ T cells. n = 30 samples. **b**, Cell type frequency calculated from total cells measured within an array. Lines represent average between duplicate arrays. Columns are separated by participant. **c**, Monocyte count from whole blood for 12 participants studied in Ndhlovu et al.^37^. Two-sided paired t test, n = 12.

To assign cellular identity, we analyzed the combined data from all participants and timepoints (Methods). These analyses yielded few participant-specific features, suggesting that disease biology, rather than technical artifact, is the main driver of variation (Fig. 1d and Extended Data Fig. 1c,d). We annotated clusters by comparing differentially expressed genes defining each to known lineage markers and previously published datasets (Extended Data Fig. 1e,f and Supplementary Table 3). These clusters recapitulate several well-established PBMC subsets (Fig. 1c), revealed phenotypic subgroupings of both monocytes (antiviral, inflammatory and nonclassical) and cytotoxic T cells (CTLs) (CD8^+^ CTL, proliferating; Extended Data Fig. 1g) and highlighted subset frequency dynamics such as natural killer (NK) cell expansion after 2–3 weeks. Flow cytometry measurements of CD45^+^CD3^+^CD4^+^ and CD45^+^CD3^+^CD8^+^ frequencies over the course of infection correlated with those measured by Seq-Well (Extended Data Fig. 2a,b and Fig. 1e). Whole blood monocyte counts, meanwhile, confirmed monocyte expansion following infection (Extended Data Fig. 2c).

**Fig. 2:**
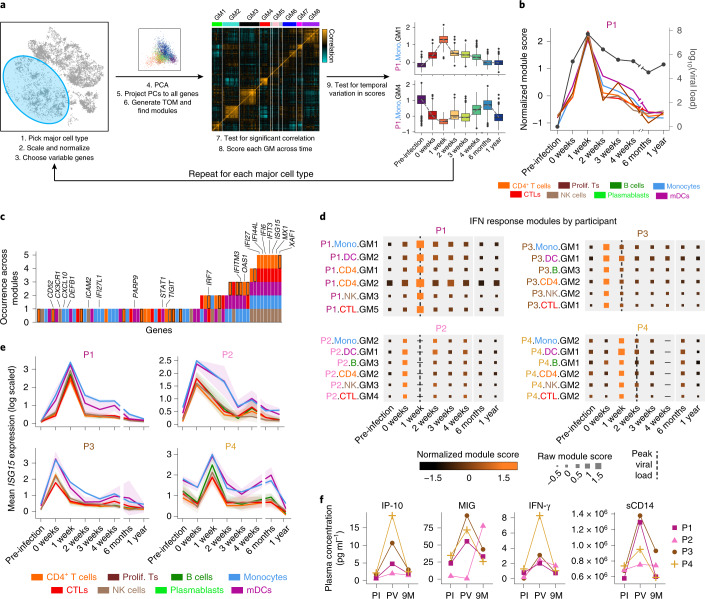
GM discovery reveals ubiquitous response to IFN with cell-type-specific features. **a**, Schema depicting temporal GM discovery (see Methods). This procedure was repeated for each major cell type (monocytes, CD4^+^ T cells, CTLs, proliferating T cells, NK cells, B cells, plasmablasts and mDCs) on a participant-by-participant basis, generating 0–8 GMs per cell type. Modules were arbitrarily numbered within a given cell type and participant. mDC, myeloid dendritic cell; PCA, principal-component analysis; PC, principal component; TOM, topological overlap matrix. **b**, In P1, six GMs across multiple cell types exhibited similar temporal expression profiles; each GM’s score (colored lines) peaked at the same timepoint as for peak viremia (line-and-dot plot). **c**, Number of occurrences of gene membership for all genes present across the six GMs in **b**. **d**, GM expression scores for IFN response modules in each participant. Normalized GM score is depicted by heat (black to orange), whereas raw module score is depicted by box size. The timepoint closest to peak viral load is indicated by a dotted line. **e**, Mean expression of *ISG15* separated by timepoint and individual. Shaded area denotes 95% CI of the mean. See Supplementary Table 2 for the number of cells per timepoint per cell type. **f**, Luminex measurements of IP-10, MIG and IFN-γ and ELISA of soluble CD14 (sCD14) in matching plasma samples. Points are averages of duplicate measurements. PI, pre-infection; PV, peak viremia; 9 M, 9 months post-detection.

Having mapped cell type frequency dynamics during acute HIV-1 infection, we next examined how different cellular phenotypes shifted over time. Previous applications of scRNA-seq to evolving cellular responses have either emphasized pseudotemporal ordering in development^9^ to delineate well-ordered progressions through cell fate^10^ or identified transcriptional differences^11^ associated with disease treatment^12^. As our dataset includes multiple, noncontiguous timepoints and complex nonlinear dynamics spaced over days to weeks, it required distinct treatment. Therefore, we developed a framework to examine how each cell type varied in phenotype over the course of infection by adapting weighted gene correlation network analysis (WGCNA) to discover, in an unbiased manner at single-cell resolution, gene modules (GMs) whose expression varied significantly over time (Fig. 2a, Methods and Supplementary Tables 4 and 5). Given the small number of participants and heterogeneity in disease response, we opted to characterize each participant and cell type independently to: 1) identify cellular responses associated with plasma viremia; 2) group modules within individuals over time; and 3) nominate molecular drivers and potential cell–cell signaling.

**Extended Data Fig. 3:**
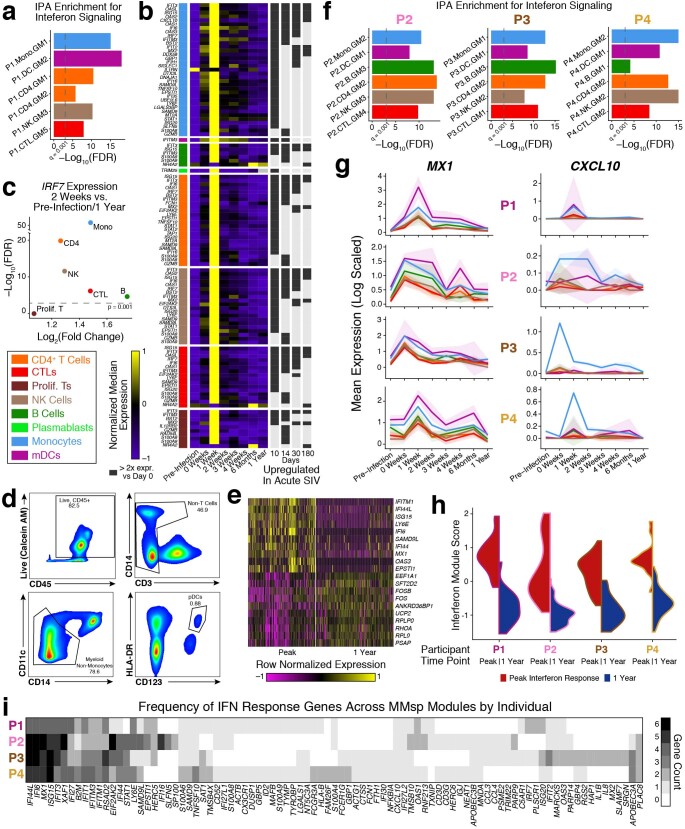
Gene modules that align near peak viremia and are differentially expressed in pDCs are enriched for response to interferon. **a**, Enrichment of modules from P1 in Fig. 2b against the IPA Interferon Signaling canonical pathway; FDR corrected Right-Tailed Fisher’s Exact Test. See Supplementary Table 4 for gene lists and their sizes. **b**, Heatmap of median expression of genes upregulated in PBMCs from SIV infection of rhesus macaques (n=8) compared to day 0 (fold change > 2) in Bosinger et al.^21^ depicted independently for each cell type in P1. Only genes differentially expressed across all time points by ANOVA in a given cell type are shown. **c**, Differential expression results for *IRF7* in each cell type (except plasmablasts and mDCs which do not have enough cells to test, n < 4) between cells from 2 weeks and pre-infection + 1 year; implemented using the “bimod” likelihood ratio test in Seurat^44^. See Supplementary Table 2 for cell numbers per cell type per timepoint. **d**, Representative gating scheme for single-cell pDC sorts. n = 8 samples. **e**, Heatmap of genes differentially expressed between pDCs captured at the same timepoints as peak interferon responses and 1-year post HIV infection. Two-sided Wilcoxon rank sum test; number of cells per timepoint: Peak – 159; 1-Year – 184. **f**, Same as **a** for the modules in Fig. 2d for P2, P3, and P4. See Supplementary Table 2 for cell numbers per cell type per timepoint. **g**, Mean gene expression (log scaled) of *MX1* and *CXCL10* over time in each individual separated by cell type. Shaded area denotes 95% confidence interval of the mean. **h**, Scoring of pDCs in each participant using a core interferon signature specific to that participant. Number of cells per participant and time point: P1 Peak – 31; P1 1-Year – 79; P2 Peak – 48; P2 1-Year – 20; P3 Peak – 42; P3 1-Year – 28; P4 Peak – 16; P4 1-Year – 57. **i**, Heatmap of gene frequency across interferon response GMs in each participant.

Within each individual, the discovered GMs demonstrated common transient patterns over the course of infection, indicating the utility of our approach in uncovering responses with shared dynamics across multiple cell types. Looking at GMs associated with changes in plasma viral load in participant 1 (P1), we identified a set of six spanning multiple cell types all sharing their highest relative module score at peak viremia (Fig. 2b). Despite being generated in distinct cell types, each GM included *IFI27*, *IFI44L*, *IFI6*, *IFIT3*, *ISG15* and *XAF1* (Fig. 2c and Extended Data Fig. 3a), in addition to other interferon (IFN)-stimulated genes (ISGs)^13^. Collectively, these expression patterns reveal cell-type-specific genes and functions correlated with a core ISG signature in P1, including monocyte antiviral activity (*CXCL10*, *DEFB1*)^14,15^, dendritic cell (DC) activation (*PARP9*, *STAT1*)^16,17^, naive CD4^+^ T cell differentiation (*CD52*, *TIGIT*)^18,19^ and NK cell trafficking (*CX3CR1*, *ICAM2*)^20^. Moreover, in P1, monocytes and DCs uniquely expressed genes (*CXCL10*, *LGALS3BP)* measured in bulk responses in acute simian immunodeficiency virus (SIV) infection in rhesus macaques^21^, which may shed light on the cellular sources of these antiviral molecules (Extended Data Fig. 3b and Methods).

Because we did not directly detect expression of IFN-I genes, potentially due to the sites or timepoints analyzed, we characterized the expression of their upstream regulator *IRF7* to infer which cell type(s) may be responsible for their production^22^. In P1, six of eight cell types studied demonstrated higher expression of *IRF7* at peak viremia compared to pre-infection and 1-year timepoints (Extended Data Fig. 3c). We also assayed plasmacytoid DCs (pDCs), which produce IFN-α and IFN-β in response to HIV^23^, at peak viremia and 1-year post-infection (Extended Data Fig. 3d,e and Methods) but did not find IFN-I gene expression or a significant change in *IRF7* expression (two-sided Wilcoxon rank-sum test, false discovery rate (FDR) corrected *q* < 1).

The three other participants studied (P2–P4) each had pDC responses and sets of ISG GMs similar to P1 at, or the week before, peak viremia, which we corroborated at the individual gene level (Fig. 2d,e and Extended Data Fig. 3f–h). Comparing GMs across individuals, we noted common ISGs (present in three or more cell types) that were shared in two or more participants (*ISG15*, *IFIT3*, *XAF1*) as well as some specific to a single participant (*APOBEC3A*, *IFI27*, *STAT1*; Extended Data Fig. 3i). To independently confirm the presence of IFNs and downstream cytokines, we measured IFN-γ, MIG (CXCL9) and IP-10 (CXCL10; previously associated with disease progression and infection outcome^24^; Fig. 2f and Methods). All participants demonstrated higher levels of IFN-γ and IP-10 at peak viremia and three demonstrated elevated MIG. We also observed increased soluble CD14, known to be associated with monocyte activation^25^.

Given concerted and cell-type-specific IFN responses during hyperacute HIV infection, we next explored whether other modules exhibited shared expression dynamics. We applied fuzzy c-means clustering to the median module scores at each timepoint across all cell types on a participant-by-participant basis, generating clusters of modules which we refer to as meta-modules (MMs) (Methods). MMs represent gene programming across distinct cell types with coordinated temporal dynamics—here, synchronized responses to infection—enabling us to link cellularly discrete but contemporaneous behaviors to both common and unique propagators.

**Extended Data Fig. 4:**
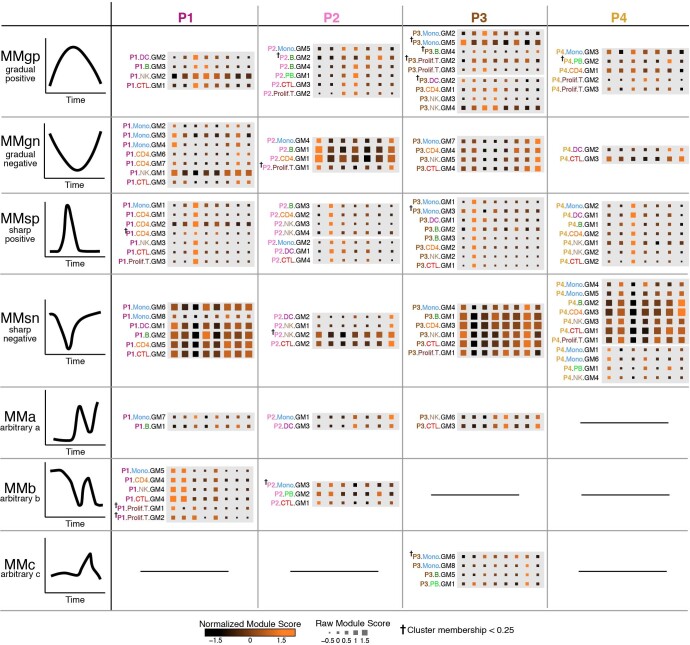
All significant temporally variant modules in all participants grouped by fuzzy c-means clustering. Modules grouped by fuzzy c-means clustering (see Methods for choice of c) reside in the same gray box. Each group of modules, or meta module (MM), were then aligned across participants based on overall temporal trend (left column). Some participants had multiple MM with similar temporal dynamics and were grouped within the same MM. Since fuzzy c-means clustering assigns membership values to each member of a cluster, we report any modules that demonstrated low cluster membership with **†**.

**Extended Data Fig. 5:**
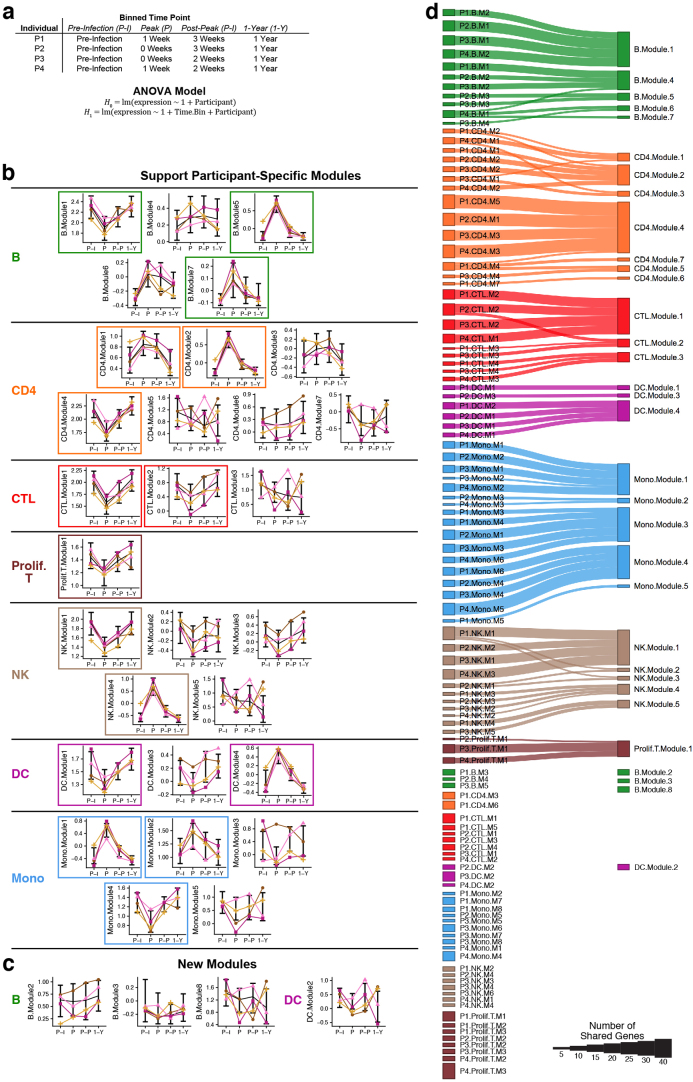
Cross-participant module discovery recapitulates aspects determined by calculating participant-specific modules. **a**, Timepoints chosen for testing significant changes in module expression over time across all participants. Timepoints were chosen based on peak expression of modules in MM1 and MM3. We used an ANOVA model to account for participant-specific features. **b**, Significant cross-participant modules that map to participant-specific modules (share at least 5 genes), separated by cell type. Median module expression is plotted for each module split by participant. Error bars depict the upper and lower quartiles for all cells across all four individuals at each time point. Boxed modules demonstrate consistent directional trends in expression between each pair of timepoints in at least 3/4 participants. P-I = pre-infection; P = peak; P-P = post-peak; 1-Y = 1 year. See **a** and Supplementary Table 2 for cell numbers per cell type per participant. **c**, Significant cross-participant modules that do not correspond to participant-specific modules. **d**, Sankey diagram demonstrating the gene overlap between participant specific modules (left) and cross-participant modules (right). Node size correlates with the number of genes within the module and edge width correlates with the number of shared genes between modules. Only overlaps consisting of ≥5 genes have edges depicted.

We next identified MMs from every participant and grouped them by their expression dynamics (Extended Data Fig. 4). We labeled four of these on the basis of their transient peak expression score patterns: sharp positive (MMsp), sharp negative (MMsn), gradual positive (MMgp) and gradual negative (MMgn); three additional MMs, labeled a–c, demonstrated more complex patterns. Besides MMsp, which contained the majority of the ISG modules, only MMsn, enriched for ribosomal protein-coding genes (Supplementary Table 4) previously shown to indicate cellular quiescence^26^, spanned five or more cell types. In parallel, we attempted to discover conserved modules across individuals, using cells from all four participants binning timepoints by viral load (Methods, Extended Data Fig. 5a and Supplementary Table 6). All but four of these cross-participant modules recapitulated those found in our participant-specific approach (Extended Data Fig. 5b–d). However, this pan-participant analysis did not reveal any GMs with consistent expression trends (in at least three out of four participants) besides the already identified ISG (MMsp) and ribosomal protein (MMsn) modules and failed to discover several participant-specific modules within MMgp.

**Fig. 3:**
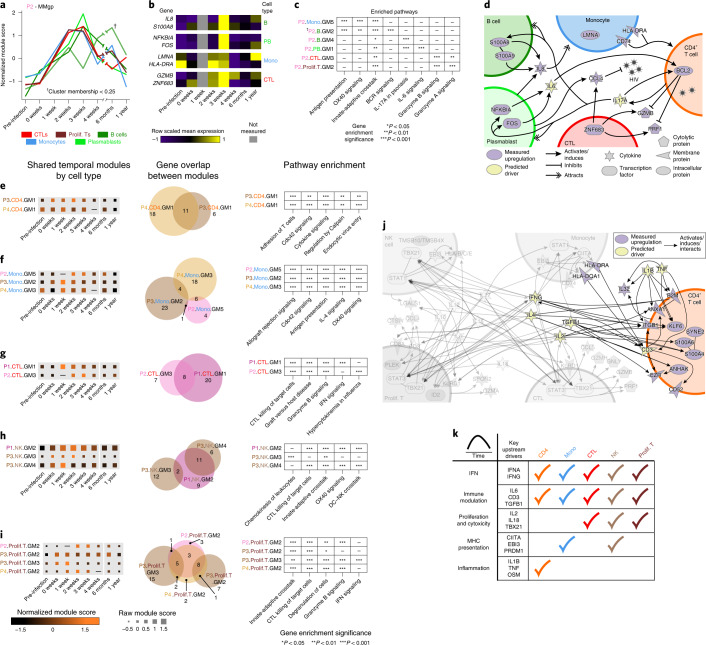
Distinct modules across different cell types share temporal expression patterns in acute HIV infection and suggest shared and cell-type-specific drivers of immune response. **a**, Normalized module expression scores for the six GMs clustered into meta modules: gradual positive (MMgp) in P2. † indicates GMs with MM membership score < 0.25 **b**, Mean gene expression of representative genes from modules in **a**; see Supplementary Table 4 for full gene lists. **c**, Select enriched pathways for the genes in each module from **a**; gene-set enrichment performed in ingenuity pathway analysis (IPA); see Supplementary Table 6 for the full list, using a right-tailed Fisher’s exact test. See Supplementary Table 4 for gene list sizes. **d**, Putative cell–cell signaling network. Nodes represent gene products with either measured gene upregulation in the modules in **a** or predicted drivers from IPA. Edges were drawn from connections nominated by IPA and curated from the literature. **e**–**i**, Module scores (left), gene overlaps between modules (middle) and enriched pathways (right; IPA) for modules grouped in MMgp and shared across participants in CD4^+^ T cells (**e**), monocytes (**f**), CTLs (**g**), NK cells (**h**) and proliferating T cells (**i**). Enriched pathways were determined using a right-tailed Fisher’s exact test. See Supplementary Table 4 for gene lists and their sizes. **j**, Putative cell–cell signaling network derived from genes shared across ≥2 participants from modules in **e**–**i**; see Supplementary Table 7. Nodes and edges are drawn as in **d**. Here we highlight those molecules interacting with or measured in CD4^+^ T cells; the full network is presented in Extended Data Fig. 7b. To reduce complexity, we omitted nodes depicting expression of *GZMB*, *PRF1* and *GNLY* by both NK cells and proliferating T cells and *CCL5* by proliferating T cells. **k**, Summary table of immune responses to related and distinct stimuli with similar temporal dynamics, defined by transient increased module expression for several weeks after peak viremia.

Notably, MMgp consisted of responses sustained throughout acute infection, but implicated different cell types in each participant. For example, in P2, MMgp consisted of monocyte, B cell, plasmablast, CTL and proliferating T cell GMs (Fig. 3a). Unlike MMsp (ISGs), these GMs spanned several distinct gene expression programs, such as antigen presentation (monocytes and B cells), interleukin (IL)-6 and IL-8 production (plasmablasts) and granzyme B production (CTLs; Fig. 3b,c and Supplementary Table 7). As these overlap in time, they may represent cell subsets responding to common stimuli and/or one another. Looking for known relationships between genes within and across cell types, we generated a network model describing potential axes of cell–cell signaling, both direct (via receptor–ligand) and indirect (signaling via chemokines and cytokines), in P2 (Fig. 3d and Methods). Expression of IL-8 and IL-6 in B cells and plasmablasts^27^ may attract monocytes presenting antigen to prime CD4^+^ T cells, potentially leading to IL-17 production^28^ and *BCL2* upregulation, known to restrict CTL-mediated killing of infected cells^29^. Together, this suggests the IL-6–IL-8–IL-17 signaling axis as a potential target for future HIV treatment.

**Extended Data Fig. 6:**
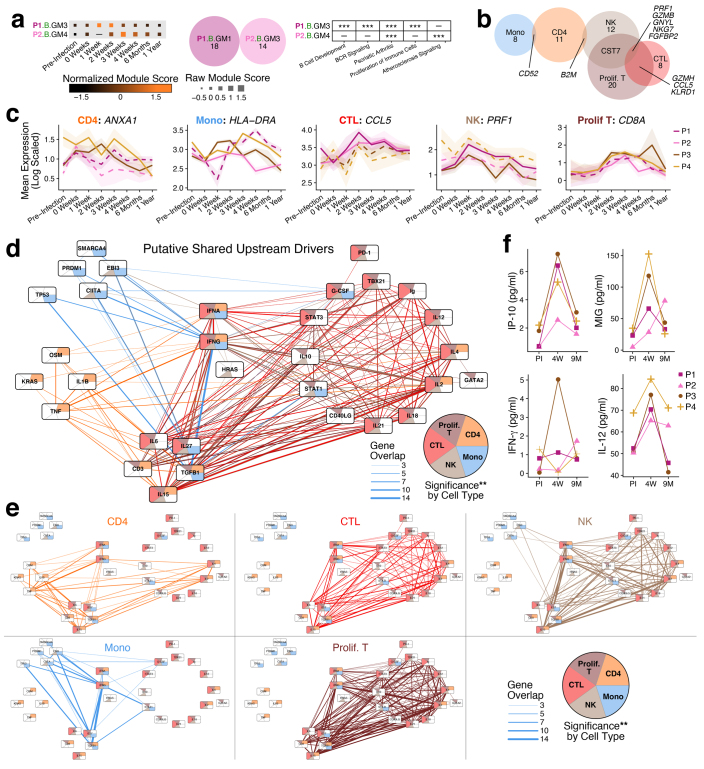
Sustained B cell modules and shared genes and upstream drivers between participants. **a**, B cell modules in MM1 with high cluster membership. **b**, Euler diagram of conserved overlapping genes between cell types from Fig. 3e–i, see Supplementary Table 8. **c**, Mean expression of *ANXA1, HLA-DRA, CCL5, PRF1*, and *CD8A* in CD4^+^ T cells, monocytes, CTLs, NK cells, and proliferating T cells, respectively. Shaded area denotes 95% confidence interval of the mean. Participants who did not have modules with shared temporal expression pattern as outlined in Fig. 3e–i are shown as dashed lines. See Supplementary Table 2 for cell numbers per timepoint per participant. **d**, Network of predicted upstream drivers of modules in Fig. 3e–i. Nodes are colored by significance in each cell-type. Edge width and color reflect the number of shared genes (width) in the gene sets of the upstream drivers for a given cell-type (color; see Methods). **e**, **d** displayed with only the edges from a given cell type. **f**, Luminex measurements of IP-10, MIG, IFN-γ, IL-12 in matching plasma samples. Points are averages of duplicate measurements. PI = pre-infection; 4W = 4 weeks post-detection; 9M = 9 months post-detection.

Given the diverse, participant-specific GMs in MMgp (Extended Data Fig. 4), we next looked whether any acute infection responses were present in multiple participants. In CD4^+^ T cells, monocytes, NK cells, CTLs and proliferating T cells, we found GMs in MMgp that shared genes in two or more participants (Fig. 3e–i; see Supplementary Table 8 for overlapping genes). While DCs and B cells also expressed multiple GMs within MMgp, some did not share any genes across participants (Extended Data Fig. 6a) or had low membership scores and were thus excluded (membership <0.25, labeled with **†** in Extended Data Fig. 4; Methods).

We next qualitatively compared GM functional annotations within MMgp for each cell type across participants (Fig. 3e–i and Methods). Despite variable temporal dynamics and unique gene memberships, we observed significant enrichment for ≥15 of the same underlying pathways and functions in at least two participants (*P* < 0.01, Supplementary Table 9), suggesting the existence of common features across individuals despite heterogeneity in infection response. For example: 1) CD4^+^ T cells (P3 + P4) expressed genes associated with nonclassical viral entry by endocytosis^30^ and adhesion, suggesting migration and viral dissemination throughout the body; 2) monocytes (P2 + P3 + P4) expressed genes associated with antigen presentation, potentially indicating generalized IFN responses or the potential to promote active T helper and CTL responses^31^; and 3) NK cells (P1 + P3), CTLs (P1 + P2) and proliferating T cells (P2 + P3 + P4) upregulated genes associated with killing of target cells by perforin and granzyme release, highlighting the joint role of innate and adaptive lymphocytes in combating viremia^32,33^ (see Supplementary Table 7 and Extended Data Fig. 6b for shared genes). Gene expression data corroborate these GM expression trends (Extended Data Fig. 6c).

We hypothesized that there may be a common set of immune drivers coordinating these gene responses during infection. To identify potential inducers of the GMs in MMgp, we generated a list of predicted upstream drivers for each. Using hits that were significant for two or more GMs, we constructed a network detailing putative upstream signaling (Supplementary Table 9, Extended Data Fig. 6d,e and Methods), highlighting potential roles for: 1) IFN-α and IFN-γ across all five cell types; 2) IL-15, IL-12 and IL-21 in CTLs, NK and proliferating T cells; and 3) IL-1β and tumor necrosis factor (TNF) restricted to CD4^+^ T cells. Parallel Luminex measurements confirmed increased IP-10, MIG and IL-12, but not IFN-γ, in plasma at 4 weeks, near when MMgp peaked in each individual (Extended Data Fig. 6f).

**Extended Data Fig. 7:**
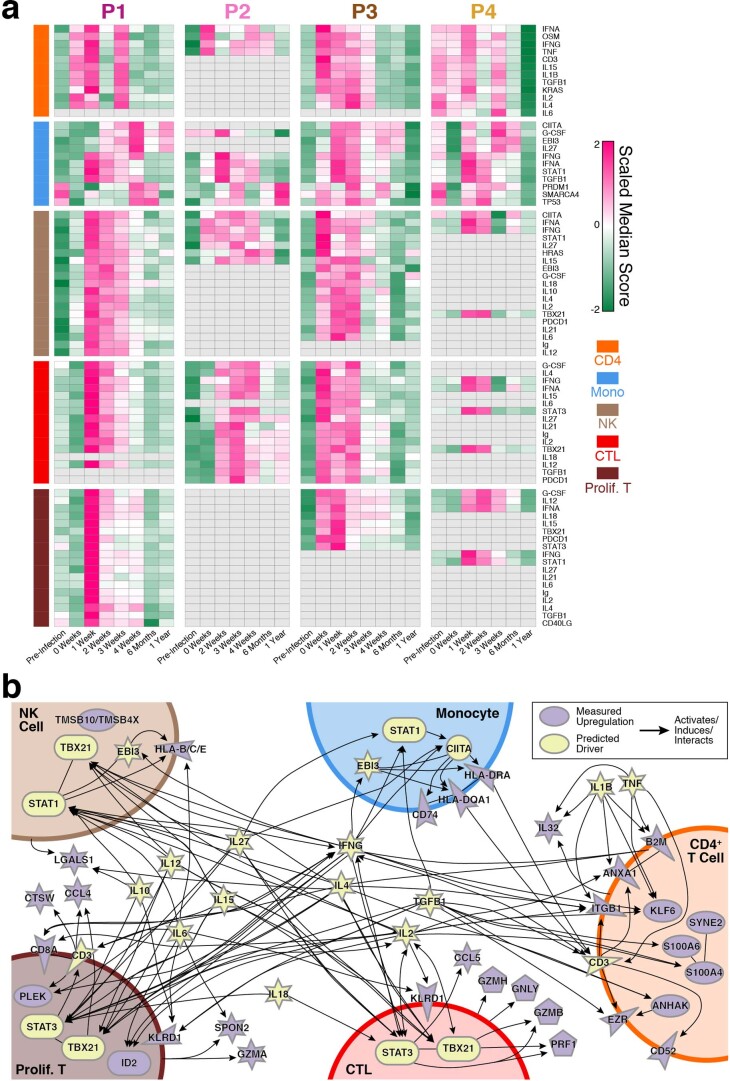
Putative upstream drivers highlight variable response dynamics and cell-cell signaling. **a**, Median gene set scores for significantly temporally variant (p < 0.05) upstream drivers in all participants. Gray boxes indicate that the upstream driver was not significantly variant in that cell type and participant. Right-Tailed Fisher Exact Test; see Supplementary Table 2 for cell numbers per timepoint per cell type. **b**, Putative cell-cell network described in Fig. 3j, but all nodes and connections depicted. Nodes represent genes with either measured upregulation in the modules in Fig. 3e–i or predicted drivers from IPA. Edges were drawn from connections nominated by IPA and curated from the literature.

Re-scoring cell types against enriched genes for each driver revealed variable kinetics in the onset, intensity and length of immune responses across different cell types (Extended Data Fig. 7a). We note the following gene-programming upregulation trends in all participants: 1) CD4^+^ T cells activity from before peak viremia throughout acute infection; 2) CTL and proliferating T cell programs are induced during hyperacute infection; and 3) NK cell and monocyte activity persists throughout the first month of infection, highlighting a persistent role for innate immunity throughout acute infection. Based on cell type, gene and functional enrichments, we summarize the shared (≥2 participants) immune responses with sustained gene expression over the course of the first month of HIV infection, their potential drivers and putative cell–cell signaling, emphasizing CD4^+^ T cells, which was the only cell type expressing genes downstream of proinflammatory cytokines (Fig. 3j,k and Extended Data Fig. 7b). Thus, our module discovery approach readily reveals immune responses and potential interactions among several cell types during acute HIV infection.

**Fig. 4:**
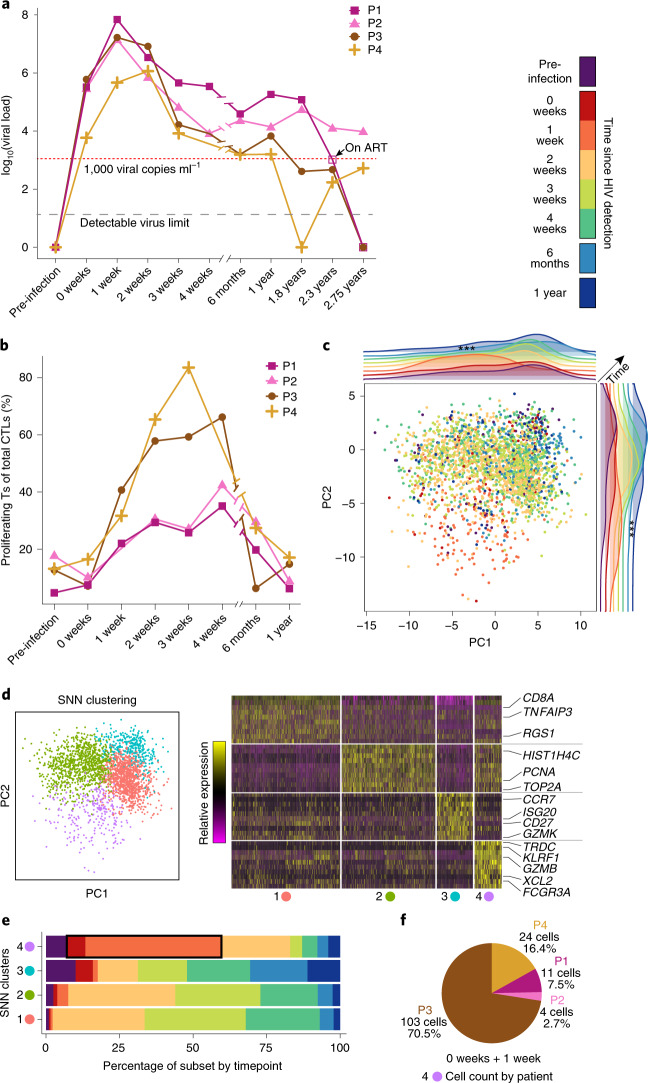
Future controllers exhibit higher frequencies of proliferating CTLs and a precocious subset of NK cells 1 week after detection of HIV viremia. **a**, Viral load by PCR with reverse transcription of the plasma of four participants assayed out to 2.75 years. Controllers of HIV maintain levels of plasma viremia <1,000 viral copies ml^−1^. P1 initiated ART before the 2.3-year timepoint. **b**, Proportion of proliferating T cells of total CTLs as a function of time and individual measured by Seq-Well. See Supplementary Table 2 for the number of cells per timepoint per cell type. **c**, PCA of proliferating T cells from all four individuals. Cells assayed from the 1-week timepoint strongly separated along PC1 and PC2; two-sample Mann–Whitney *U*-test; 174 cells (1 week) versus 2,465 cells (all other timepoints); ***P < 2.2 × 10^−16^. **d**, Shared-nearest neighbor (SNN) clustering over the top six PCs reveals four subclusters (left) with distinct gene programs (right). Two-sample Wilcoxon rank-sum test was used for analysis; numbers of cells per cluster: 1–1,081; 2–929; 3–359; 4–270. **e**, Percentage of cells in each subcluster by timepoint. **f**, Number of cells from each individual within the cells sampled at 0 weeks and 1 week in the NK cell cluster (4, lilac; black box in **e**).

**Extended Data Fig. 8:**
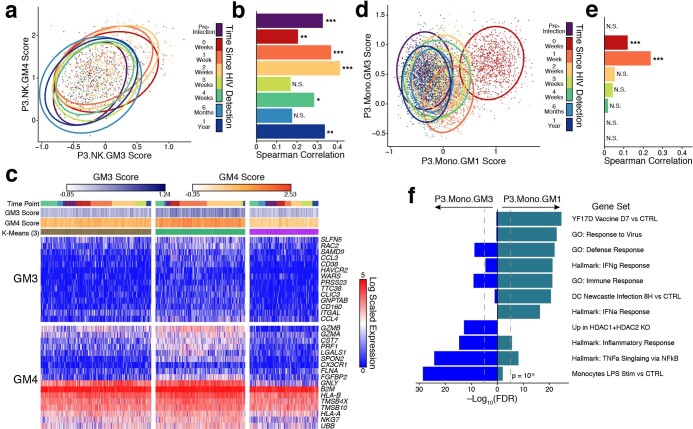
Two cases of similar temporal modules in NK cells and monocytes: variable correlation and variable co-expression. **a**, Module scores in NK cells for NK GM3 and NK GM4 in P3. Ellipses drawn at 95% confidence interval for cells from each timepoint. See Supplementary Table 2 for cell numbers per timepoint. **b**, Correlation (spearman’s rho) between the scores for NK GM3 and NK GM4 at each timepoint. Two-sided asymptotic t approximation; FDR corrected q-value: N.S = not significant; *q < 0.05; ** q < 0.01, *** q < 0.001. **c**, Gene expression heatmap of all NK cells in P3 for those genes in NK GM3 and NK GM4. Cells are separated based on k-means (k=3) over the depicted genes. **d** & **e**, Same as in **a** & **b** but for monocyte modules Mono GM1 and Mono GM3 in P3. **f**, Gene set enrichment analysis of the genes in Mono GM1 and Mono GM3 against the following MSigDB collections: Hallmark, C2, C3, C5, and C7. FDR corrected hypergeometric test; number of genes: GM1 - 33, GM3 - 52.

In our analyses, we observed GMs that demonstrated similar temporal response patterns within the same cell type but distinct pathway enrichments, implying orthogonal biological functionality: for example, the NK cytokine signaling GM3 module (*CCL3*, *CCL4*) and the cytotoxic GM4 module (*PRF1*, *GZMB*) in P3 (Fig. 3h). To understand how these GMs might be linked, we looked across single cells for module coexpression (obscured in bulk approaches). Surprisingly, the strength of the correlation between expression of these modules across single NK cells changes with time, decreasing later in infection (Extended Data Fig. 8a,b). K-means clustering separated cells by variable expression of GM3 and GM4 (Extended Data Fig. 8c). Variation in the correlation of GM3 and GM4 may reflect NK cell plasticity with dual cytotoxic and signaling programming near peak viremia.

**Extended Data Fig. 9:**
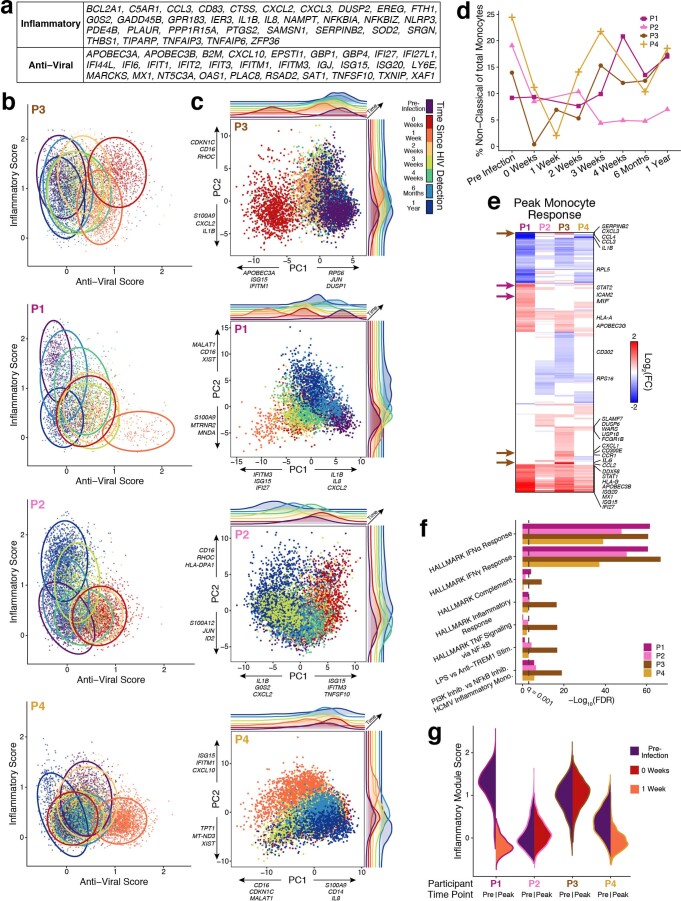
Participants demonstrate diverse monocyte responses prior to and immediately after HIV detection, wherein one participant who goes on to control infection presents a poly-functional subset of monocytes. **a**, Inflammatory and anti-viral genes shared between participants (present in at least two modules. **b**, Inflammatory and anti-viral scores of monocytes in each participant using gene lists in **a**. Ellipses drawn at 95% confidence interval for cells from each timepoint. See Supplementary Table 2 for cell numbers per timepoint per cell type. **c**, Principal component analysis (PCA) of all monocytes from each participant. Density of cells in PC1 vs PC2 space annotated by timepoint are depicted, and 3/5 of the top loading genes for PC1 and PC2 are also annotated. **d**, Percent of Non-Classical (CD16^+^/*FCGR3A*) monocytes of total monocytes as a function of time in each participant. Percentage calculated from cluster assignment (see Extended Data Fig. 1d). **e**, Heatmap of differentially expressed genes (FDR corrected q < 0.05 in at least one participant) between monocytes at the peak response timepoint (0 weeks/1 week) vs. pre-infection. Arrows indicate genes specific to P3 (dark-brown) and P1 (violet). Two-sided Wilcoxon rank sum test; see Supplementary Table 2 for cell numbers per timepoint per participant. **f**, Enriched pathways for the differentially expressed genes in **e**, using the MSigDB Hallmark Gene Sets. Hypergeometric test; FDR corrected q values; number of differentially expressed genes: P1 – 1,350; P2 – 436; P3 – 857; P4 – 514. **g**, Violin plot of the Inflammatory Module Score (see **a** for genes in the module) for monocytes at pre-infection (Pre) and peak transcriptional response time points (Peak) in each participant. See Supplementary Table 2 for cell numbers per timepoint per participant.

Examining MMsp (ISG GMs), we also observed that P3 exhibited temporally similar modules in monocytes (GM1 and GM3); however, these did not variably correlate over time. Instead, they were highly coexpressed, but only at HIV-detection (Extended Data Fig. 8d,e). Gene-set analysis demonstrated that monocyte GM1 consisted of antiviral response genes, while GM3 was enriched for genes associated with inflammation (Extended Data Fig. 8f). Thus, monocytes in P3 at the time of HIV detection are simultaneously expressing both antiviral and inflammatory gene programs, a previously unappreciated phenotype. While both gene programs strongly contributed to the major axes of monocyte variation in all individuals, we were unable to identify polyfunctional monocytes in the other participants (Extended Data Fig. 9a–c). Meanwhile, nonclassical monocytes displayed disparate temporal dynamics across participants (Extended Data Figs. 1d and 9d). Comparing differentially expressed genes at peak response timepoints (1–2 weeks) further highlighted other participant-specific differences: monocytes in all participants produced antiviral factors (Extended Data Fig. 9e,f), but only P2 and P3 were enriched for inflammatory responses and only P3 for TNF signaling via NF-κB (*q* < 0.001). Chronic inflammation has been associated with susceptibility to infection^34^ and our data show variable inflammatory gene expression before infection with subsequent mixed expression changes in hyperacute infection across participants (Extended Data Fig. 9g).

As we have previously demonstrated that natural control of HIV is associated with diverse cellular phenotypes in CTLs^35^ and DCs^4^, we looked to see whether the presence of polyfunctional monocytes in P3 might link to disease progression in chronic infection. We observed that both P3 and P4 maintained low levels of viremia (<1,000 viral copies ml^−1^) at 2.75 years after infection in the absence of antiretroviral therapy (ART) (Fig. 4a). HIV infected persons who naturally maintain low levels of viremia in chronic infection (HIV controllers) demonstrate enhanced immune responses systemically^4,36^. However, whether early events in acute HIV infection reflect or contribute to control of chronic viremia is unknown.

**Extended Data Fig. 10:**
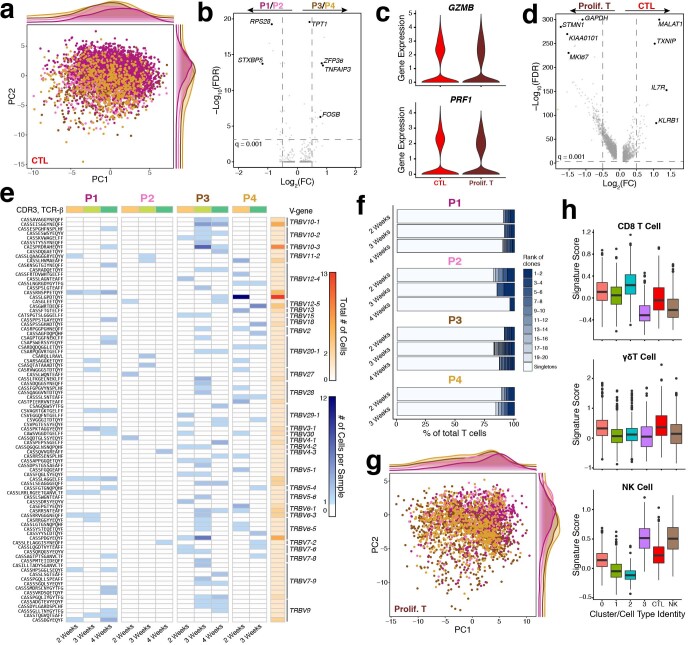
Non-proliferating and proliferating cytotoxic T cells. **a**, Principal component analysis of non-proliferating CTLs with participant density annotated along PC1 and PC2. Number of cells: P1 – 1828; P2 – 968; P3 – 1503; P4 – 670. **b**, Volcano plot of differentially expressed genes between the participants who control (P3/P4) and those who do not (P1/P2); implemented using a two-sided Wilcoxon rank sum test. See **a** for cell numbers. **c**, Expression of *GZMB* and *PRF1* in all CTLs and proliferating T cells. Number of cells: CTL – 4,969; Proliferating T cells – 2,639. **d**, Volcano plot of differentially expressed genes between non-proliferating CTLs and proliferating T cells; implemented using a two-sided Wilcoxon rank sum test; see **c** for cell numbers. **e**, Heatmap of detected TCR-β CDR3s in proliferating T cell clusters 0 & 1 at 2 weeks, 3 weeks, and 4 weeks post-HIV detection. See Supplementary Table 10 for full list of CDR3s. **f**, Distribution of ranked TCR-β CDR3 clones (by total cell number) and singletons measured from all T cells (i.e. CD4^+^ T cells, CTLs, and proliferating T cells) detected at 2 weeks, 3 weeks, and 4 weeks post-HIV detection in at least two single cells in each participant. Here, except for the singletons, each sliver represents the percentage of CDR3s ascribed to the top n-n+1 clones for that timepoint and participant. **g**, Same as in **a** but over proliferating T cells. Number of cells: P1 – 483; P2 – 273; P3 – 1193; P4 – 690. **h**, CD8 T cell (top), γδT cell (middle), and NK cell (bottom) scores for each proliferating T cell cluster (see **g** for cell numbers) and 500 randomly sampled CTLs, and 500 randomly sampled NK cells. Signatures were established from differential expression over the single-cell dataset published by Gutierrez-Arcelus et al.^46^. See Supplementary Table 10 for all differentially expressed genes and signature score gene lists. Box plots features depict: minimum = 25^th^ percentile – 1.5 * inter-quartile range (IQR; smallest value within); lower = 25^th^ percentile; center = 50^th^ percentile; upper = 75^th^ percentile; maximum = 75^th^ percentile + 1.5 * IQR (largest value within).

As CD8^+^ T cells are known to contribute to controlling chronic HIV infection^35,36^, we also analyzed CTLs from all participants, noting increasing levels of *PRF1* and *GZMB* during acute infection (Fig. 3g). Further unsupervised and directed approaches did not demonstrate significant differences in CTL responses across participants (Extended Data Fig. 10a,b and Supplementary Table 10). In FRESH, we demonstrated that the majority of proliferating CTLs in acute infection are HIV specific^37^. Therefore, we looked for differences in proliferating T cell responses by participant. On average, proliferating T cells expressed similar levels of cytotoxic genes as non-proliferating CTLs. Differential expression analysis highlighted genes associated with cell cycle and memory for proliferating and nonproliferating CTLs, respectively (Extended Data Fig. 10c,d and Supplementary Table 10). T cell receptor (TCR) pulldown and enrichment (TCR-β CDR3) revealed few expanded clones (Extended Data Fig. 10e,f, Supplementary Table 10 and Methods); this, however, may be affected by sample size (CDR3s were detected in 982 proliferating T cells). Relative to P1 and P2, both controllers (P3 and P4) displayed higher frequencies of proliferating cytotoxic cells within the first month of infection compared to pre-infection (Fig. 4b).

We next used unsupervised analyses to examine differences in proliferating T cell responses over time among participants (Fig. 4c and Extended Data Fig. 10g). Clustering over all proliferating T cells, we identified four subsets of cells with distinct gene programs (Fig. 4d and Supplementary Table 10): traditional CD8^+^ T cells, hyperproliferative CD8^+^ T cells, naive CD4^+^ T cells and a subset of cells that were *CD8A*^–^ but *TRDC*^+^ and *FCGR3A*^+^ (CD16). Using signatures from a single-cell study of cytotoxic cells, we determined that the *FCGR3A*^+^ cells were NK cells (Extended Data Fig. 10h and Methods). Looking at the distribution of cells within each of these clusters, the NK cluster contained the highest proportion of proliferating cells at HIV detection and 1 week thereafter (Fig. 4e,f). The majority of these were from P3 and P4. Thus, our data show that the two participants who maintain viral loads <1,000 viral copies ml^−1^ at 2.75 years after infection without ART exhibit a subset of proliferative, cytotoxic NK cells during the earliest stages of acute infection before the majority of HIV-specific CD8^+^ T cells arise.

Here, we present and apply a novel scRNA-seq-based framework to a unique longitudinal study of human infection in order to characterize conserved immune response dynamics, as well as cell subsets and gene programs with potential therapeutic and preventative applications. By analyzing hundreds of cells per timepoint and cell type, we were powered to identify significant changes in abundant cellular phenotypes over time in each participant; however, we could not account for all potential sources of cellular heterogeneity or their impact on infection outcome. Nevertheless, we discovered interrelated temporal GM expression patterns in distinct cell types and nominated mechanisms by which multiple components of the immune system may respond collectively—sometimes with different gene programs—to HIV infection. By identifying upstream drivers that may induce our MMs, we hypothesized when and how various cytokines, chemokines and transcription factors might orchestrate immune responses during infection. Together, our work affords a unique reference dataset for studying the earliest moments of HIV infection after detection and suggests potentially new roles for monocytes, NK cells and CD4^+^ T cells in acute infection.

Our single-cell approach also enabled us to identify cellular subsets present during hyperacute HIV infection in two individuals (P3 and P4) who maintained low viremia in chronic infection. In addition to polyfunctional monocytes identified in P3, we found a subset of cytotoxic, proliferating NK cells in P3 and P4. In other infection settings^38,39^, NK cells have demonstrated antigenic memory, suggesting that these cells could be responding to some previously encountered antigen; however, while all participants tested negative for sexually transmitted infections pre-HIV-infection, we did not screen for other chronic infections or exposures. These proliferating NK cells may function alongside CTLs early in infection, mitigating CTL antigenic load and subsequent exhaustion^40^. Although there are ethical and practical difficulties associated with collecting additional samples from untreated HIV infected persons, follow-up studies in nonhuman primates could determine the functional, actionable importance of early immune responses for long-term viral control. Future work in FRESH will seek to test the effects of early administered ART on longitudinal HIV response dynamics. Parallel efforts in human cohorts with other viral and bacterial infections, as well as inflammatory diseases and cancers, will enable assessment of the broad utility of the framework described herein, and begin to reveal the common and unique immune response motifs that inform human immunity for future modulation with drugs or treatments.

## Methods

### Study participants

All participants in this study were enrolled in the FRESH cohort^5,41^. This prospective study recruits women who are HIV negative, aged 18–24 years and are tested for HIV-1 RNA in plasma twice weekly for 1 year. Each time the women come to the study center, they participate in peer-support groups and receive a stipend. In addition to semi-weekly virus testing by PCR with reverse transcription, whole blood is collected four times (including during enrollment) throughout the year from participants. If a plasma test comes back positive, the participant is asked to come back to the clinic that day to collect a blood sample. Samples are then collected weekly through the first 6 weeks of infection and regularly afterward as long as the participant continues to return to the study center. In the arm of the study described herein, participants were initiated on ART when their CD4 count fell below 350 cells µl^−1^, per standard treatment guidelines at the time of enrollment. A second arm of the study was initiated in 2014 and is currently still in place; in that arm, participants who test positive for viral RNA are initiated on ART when they are called back into the study center for their first post-infection sample collection. To the best of our knowledge, all participants in this study had not yet started ART for the timepoints processed here. FRESH was performed in accordance with protocols approved by the Institutional Review Board at Partners (Massachusetts General Hospital), MIT and the Biomedical Research Ethics Committee of the University of KwaZulu-Natal. All FRESH participants consented for genetic and genomic data collection and analysis.

### Cell preparation, flow cytometry and cell sorting

The Life Sciences Reporting Summary contains information on the sample preparation, antibodies, gating strategy and sort strategy used in this study.

### Single-cell RNA-seq with Seq-Well

The Seq-Well platform was utilized as previously described^42^ to capture the transcriptomes of single cells on barcoded mRNA capture beads. In brief, 10 µl of sorted CD45^+^Calcein Blue^+^ PBMCs were mixed at 1:1 dilution with Trypan blue and counted using a hemocytometer. The cells were resuspended in RPMI + 10% FBS at a final concentration of ~100,000 live cells ml^−1^ and 20,000–25,000 cells in 200 µl were added to each Seq-Well array preloaded with barcoded mRNA capture beads (ChemGenes). Two arrays were used for each sample to increase cell numbers. The arrays were then sealed with a polycarbonate membrane (pore size of 0.01 µm), cells were lysed, transcripts were hybridized to the beads and the barcoded mRNA capture beads were recovered and pooled for reverse transcription using Maxima H-RT (Thermo Fisher EPO0753) and all subsequent steps. After an Exonuclease I treatment (NEB M0293L) to remove excess primers, whole transcriptome amplification (WTA) was carried out using KAPA HiFi PCR Mastermix (Kapa Biosystems KK2602) with 2,000 beads per 50 µl of reaction volume. Libraries were then pooled in sets of eight (totaling 16,000 beads) and purified using Agencourt AMPure XP beads (Beckman Coulter, A63881) by a 0.6× volume wash followed by a 0.8× volume wash and quantified using Qubit hsDNA Assay (Thermo Fisher Q32854). Quality of the WTA product was assessed using the Agilent hsD5000 Screen Tape System (Agilent Genomics) with an expected peak >800 bp tailing off to beyond 3,000 bp, and a small or non-existent primer peak, indicating a successful preparation. Libraries were then constructed using a Nextera XT DNA library preparation kit (Illumina FC-131-1096) on a total of 750 pg of pooled cDNA library from 16,000 recovered beads using index primers as previously described^43^. Tagmented and amplified sequences were purified using a 0.8× volume AMPure XP bead wash yielding library sizes with an average distribution of 500–750 bp in length as determined using an Agilent hsD1000 Screen Tape System (Agilent Genomics). Two Seq-Well arrays were sequenced per NextSeq500 sequencing run with an Illumina 75 Cycle NextSeq500/550 v2 kit (Illumina FC-404-2005) at a final concentration of 2.4 pM. The read structure was paired end with Read 1, starting from a custom read 1 primer, covering 20 bases inclusive of a 12-bp cell barcode and 8-bp unique molecular identifier (UMI), then an 8-bp index read and finally Read 2 containing 50 bases of transcript sequence.

### Seq-Well alignment, cell identification and cell type separation

Read alignment, cell barcode discrimination and UMI per transcript collation were performed as by Ordovas-Montanes et al.^43^ using a hg19 reference. Initially, we aligned the sequences from P1 to a combined HIV + hg19 genome using the consensus sequence of HIV clade C viruses from the HIV Sequence Database (https://www.hiv.lanl.gov/content/sequence/HIV/mainpage.html). After alignment, however, we measured 0–2 cells with HIV transcript alignments per array; therefore, we used the standard hg19 reference for our analysis. UMI-collapsed data were used as input into Seurat^44^ (v.2.3.4) for cell and gene trimming and downstream analysis. The following steps were performed on all of the arrays processed from a single participant, on a participant-by-participant basis. Any cell with <750 UMIs or >6,000 UMIs (0–5 cells per array) and any gene expressed in fewer than five cells were discarded from downstream analysis. This cells-by-genes matrix was then used to create a Seurat object for each participant. Cells with >20% of UMIs mapping to mitochondrial genes were then removed (50–100 cells per array). These objects (one per participant) were then merged into one object for pre-processing and cell type identification

The combined Seurat object was log-normalized with a size factor of 10,000 and scaled without centering. Additionally, linear regression was performed to remove unwanted variation due to cellular complexity (nUMI) and low-quality cells (percent.mito). Subsequently, 3,251 variable genes were identified using the ‘LogVMR’ function and the following cutoffs: x.low.cutoff = 0.01, x.high.cutoff = 10 and y.cutoff = 0.25. PCA was performed over these genes and the top 17 PCs were chosen for clustering and embedding on the basis of the curve of variance described by each PC and the genes most contributing to each PC. Next, FindClusters (SNN graph + modularity optimization) with a resolution of 0.5 was used to generate 13 clusters and the Fourier transform *t*-distributed stochastic neighbor embedding (*t*SNE) implementation^45^ with 2,000 iterations to embed the data into two-dimensional space.

Cluster identity was assigned by finding differentially expressed genes using Seurat’s implemented Wilcoxon rank-sum test and then comparing those cluster-specific genes to previously published datasets^46–48^. One cluster exhibited no cluster-specific genes; the cells from this cluster were embedded centrally in the *t*SNE, and on further investigation expressed both myeloid and lymphocyte markers. Therefore, these cells were removed as multiplets (when multiple cells enter the same well in the Seq-Well array). After multiplet removal, 59,162 cells were captured across all samples processed. The remaining 12 clusters included subsets of major circulating immune cells (see Supplementary Table 3 for marker genes). These clusters were merged by parent cell type (T cell, cytotoxic T cell, B cell, plasmablast, DC and monocyte) for downstream analysis, as variation in the SNN graph parameters weakly affected cluster assignment to the subsets.

As NK cells share many markers transcriptionally with cytotoxic T cells^46^, clustering in our dataset did not separate these two cytotoxic cell types. NK cells were annotated based on lacking expression of CD3 (*CD3D*, *CD3E*, *CD3G*) and nonzero expression of CD16 (*FCRG3A*) and *KLRF1*. CD56 (*NCAM*) was not highly expressed in our data and therefore was not used to separate NK cells. Any cell with a cluster identity belonging to the cytotoxic T cell cluster that lacked CD3 expression or expressed CD16/*KLRF1* was annotated as an NK cell. With this annotation, we noted distinct transcriptional responses between NK cells and CTLs both as a function of time and gene membership (Fig. 2c and Fig. 3g,h).

For downstream analysis of temporal variation in expression, the dataset was separated by participant and cell type: CD4^+^ T cells, NK cells, CTLs, proliferating T cells, B cells, plasmablasts, mDCs and monocytes. The expression matrix and associated metadata can be accessed online through the Single Cell Portal hosted by Broad Institute of MIT and Harvard (see Data Availability; https://singlecell.broadinstitute.org/single_cell/study/SCP256).

### Cell type normalization

Once separated by cell type and participant, the single-cell transcriptomes were processed on a cell-type-by-cell-type basis across all timepoints. For each cell type, the presence of residual contaminant RNA or doublets was assayed by scoring every cell against a set of contaminant genes from other cell types built from our marker list used to discern cluster identity (see Supplementary Table 11 for cell-type-specific contaminant gene lists and cutoffs). Cells with high contamination scores (0–10% of cells) were subsequently removed from further analysis to avoid unwanted variation in the subsequent unsupervised module discovery. Following contamination filtering, data underwent scaling and normalization, followed by variable gene discovery (~400–1,000 genes, dependent on cell type and cell number). PCA was then applied on the limited set of genes, followed by projection to the rest of the genes in the dataset.

### Module discovery

For the module analysis, we subset our data on the top and bottom 50 genes, after projection, for the first 3–9 PCs (dependent on the variance described by each PC and genes contributing to each PC) as input for WGCNA^49,50^ functions. Following the WGCNA tutorial (https://horvath.genetics.ucla.edu/html/CoexpressionNetwork/Rpackages/WGCNA/Tutorials/), an appropriate soft-power threshold was chosen to calculate the adjacency matrix. As scRNA-seq data is impacted by transcript dropout (failed capture events), adjacency matrices with high power further inflate the impact of this technical limitation and yield few correlated modules. Therefore, when possible, a power was chosen as suggested by the authors of WGCNA (that is, the first power with a scale-free topology >0.8); however, in instances where this power yielded few modules (fewer than three), we decreased our power. As a rule, smaller soft powers lead to fewer large-sized modules, whereas larger soft powers lead to more small-sized modules. In our analysis, there was frequently a distinct tipping point where, as we increased soft power, modules would fail to be identified by WGCNA (due to low connectivity). We ran our analyses with several soft powers to find an appropriate balance to generate a maximal number of modules without losing GM membership. Next, an adjacency matrix was generated using the selected soft power and it was transformed into a TOM. Subsequently, this TOM was hierarchically clustered and the cutreeDynamic function with method ‘tree’ was used to generate modules of correlated genes (minimum module size of ten). Similar modules were then merged using a dissimilarity threshold of 0.5 (that is, a correlation of 0.5); WGCNA typically suggests dissimilarity thresholds of 0.8–0.95, but we sought to avoid any spurious cluster separation potentially associated with the chosen soft power.

To test the significance of the correlation structure of a given module, a permutation test was implemented. Binning genes in the true module by average gene expression (number of bins was ten), genes with the same distribution of average expression from the total list of genes used for module discovery were randomly picked 10,000 times. For each of these random modules, a one-sided Mann–Whitney *U*-test was performed to compare the distribution of dissimilarity values between the genes in the true module and the distribution of dissimilarity values between the genes in the random module. Correcting the resulting *P* values for multiple hypothesis testing by Benjamini–Hochberg FDR correction, a module was considered significant if <500 tests (*P* < 0.05) had FDR > 0.05. We note that if we chose a smaller soft power for TOM generation, which in turn resulted in larger modules with fewer excluded genes, fewer modules passed this permutation test, likely due to noisier genes that maintained weak correlations with all other genes in the analysis.

Since we were interested in identifying modules of genes that changed in expression as a function of time, another permutation test was implemented to identify modules that significantly vary from pre-infection. First, every cell was scored for the genes within the module, using the AddModuleScore function in Seurat. This function calculates an average module score by calculating the mean expression of the genes within the module corrected for expression of other genes with similar means across the dataset. Thus, this score functions as an expression estimate of the genes within a module in any given single cell. As testing for differences in distribution is sensitive to sample number, a sample size (*s*) was selected based on the number of cells present at any given timepoint within a cell type. The smallest *s* used was ten; this cutoff was chosen based on the least frequent cell types having ~100 cells total across all timepoints within a participant. If a timepoint had fewer than ten cells, that point was not used in the testing. In the case of plasmablasts and mDCs in multiple participants, more than three timepoints had fewer than ten cells and therefore no modules were considered significantly variant in time. To determine whether module expression varied over time, 1,000 two-sided Mann–Whitney *U*-tests between the distribution of scores from *s* random cells at pre-infection and *s* random cells from each other timepoint were performed. For each timepoint, the *P* values from the 1,000 tests were averaged. After FDR correction, if *q* < 0.05 for any timepoint, the module was considered to significantly vary in expression in time. Our approach and tests have been written as functions in R and have been included as Supplementary Software.

While a similar approach is possible using bulk RNA-seq data, here, we are powered to identify temporally similar modules active in distinct subsets of cells both within and across time and we can use each cell of a specific type as a well-controlled, independent biological replicate to identify, from a single sample, essential response features and their putative upstream drivers. Compared to a directed approach, this discovery-based identification of temporally variant modules enables unbiased selection of coordinated genes and pathways and immediately reveals differences in response dynamics among cell types, states and participants.

For the cross-participant module discovery analysis (Extended Data Fig. 5), we applied the WGCNA framework to all cells of a given cell type across all four participants at all timepoints sampled. Here, the number of genes input into the framework varied between ~350–850 genes by choosing the top and bottom 100 genes from the most significant PCs, determined by finding the asymptote in the PC elbow plot (ranked s.d. of each PC). These modules were then tested for significant correlation against random sets of genes using the same permutation test outlined above. To test for temporal variability in module expression across all four participants, we binned timepoints into pre-infection, peak, post-peak and 1-year and implemented an analysis of variance (ANOVA) across binned timepoints accounting for participant heterogeneity (see Extended Data Fig. 5a). Specifically, we fitted a linear regression to the data across binned timepoints using two models: (1) null hypothesis ~1 + participant; and (2) alternative hypothesis ~1 + participant + time.bin. We then calculated the *F* statistic for the ANOVA between these two models. Peak and post-peak timepoints were chosen based on the score maxima for the modules discovered in each participant in MMsp and MMgp (see Extended Data Fig. 4).

### Module grouping and gene-set analysis

To more easily compare modules by temporal pattern within and between participants, fuzzy c-means clustering was applied to all of the modules in a given participant using the Mfuzz package^51^ (v.2.38.0). We chose to use fuzzy c-means clustering to allow us to understand the extent of membership of a given module to its assigned cluster. For each participant, c was chosen to be 5–7, such that diverse temporal patterns were separated, minimizing the number of clusters containing fewer than three modules. These groupings of modules were then annotated by similar scoring patterns across participants, taking into consideration that infection time is not the same for every participant (Extended Data Fig. 4). We named four of these MMs on the basis of the transient module score dynamics of each: MMsp, MMsn, MMgp and MMgn. The remaining three MMs were named a–c given their more complex score dynamics.

Gene-set analysis on modules was performed using IPA (Qiagen) given its better performance with low gene numbers; our modules were sized between 10–66 genes. Only gene names were supplied for analysis and submitted for core analysis with the experimentally observed confidence setting. In Fig. 3d–i, the pathways annotated were taken from either the canonical pathways or diseases and functions results. For the upstream driver analysis in Extended Data Fig. 6d,e, upstream drivers were selected by the following criteria: significant (*P* < 0.001) in at least two modules of any given cell type, with at least five genes in the gene set. As the gene sets annotated in IPA are quite large and share many genes, the edges in our network were restricted to only those upstream drivers that shared three or more genes. To achieve finer grain temporal resolution on putative inducers of immune response, the union of enriched genes for each upstream driver from modules within a given cell type was used to generate scores against the single-cell expression data. Only upstream driver scores that demonstrated temporal variability (as described above) were included. We report the median scores at each timepoint for each upstream driver.

We chose to use parts of MSigDB v.6.2 (http://software.broadinstitute.org/gsea/msigdb) for the gene-set enrichment analysis in Extended Data Figs. 8 and 9, given higher gene numbers (>100), allowing for more conservative *P* values. Multiple-hypothesis testing was corrected by the Benjamini–Hochberg FDR procedure. The specific collections of gene sets used are reported in the figure legends.

### Cell–cell signaling network curation

The cell–cell signaling networks in Fig. 3d,j and Extended Data Fig. 7b were generated using connections annotated in IPA^52^. Molecules of interest were chosen from genes in the modules belonging to MMgp in P2 (Fig. 3d) or shared among at least two participants (Fig. 3j, Extended Data Fig. 7b and Supplementary Table 7), respectively. We also included select upstream drivers found to be significant by IPA given enrichment of downstream genes within the modules. Edges were drawn between all nodes (genes or predicted upstream drivers) with the ‘Connect’ tool in ‘My Pathways’ using both ‘Direct’ and ‘Indirect’ interactions. Subsequently, edges were manually trimmed by looking at the provided support for the connections and discarding any connections not supported by demonstrations of expression or activation in the literature. For Fig. 3j, any predicted upstream driver–gene edge that connected to a cell for which that upstream driver was not significantly enriched was also trimmed (for example, only edges between IL-1β and nodes for genes in CD4^+^ T cells were kept). Contextualization of these cell–cell signaling networks is further explored online: http://shaleklab.com/resource/immune-dynamics-of-acute-hiv-infection.

### Comparison to microarray data generated in acute SIV infection

To contextualize our single-cell results against previously published datasets in SIV infection, we compared genes differentially expressed at peak viremia (compared to pre-infection) in all eight cell types studied in P1 to genes upregulated in rhesus macaques 0–180 d after SIV infection^21^. Any genes found to be differentially expressed (FDR-corrected *q* < 0.05) in our data are depicted alongside any gene that demonstrated log_2_(fold change) upregulation at any timepoint in the Bosinger et al. microarray experiments^21^ (Extended Data Fig. 3b). We note that compared to many of the ISGs upregulated in acute SIV infection, where upregulation persists for >2 weeks, most ISGs in P1 were only differentially expressed at the 1-week timepoint, indicating potential differences in the evolution of immune responses between humans and macaques.

### scRNA-seq of pDCs with SMART-seq2 and analysis

Reverse transcription, WTA and library preparation of single pDCs in 96-well plates was performed as previously described^53^. Samples were sequenced on an Illumina NextSeq500/550 instrument with an Illumina 75 Cycle NextSeq500/550 v2 kit (Illumina FC-404-2005) using 30-bp paired-end reads. Given difficulties acquiring pDCs from pre-infection samples due to limited cell numbers, we sequenced pDCs from the peak IFN response and the 1-year timepoints in each participant. Reads (5 × 10^5^ to 3 × 106 per cell) were aligned to the hg38 (GENCODE v.21) transcriptome and genome using RSEM^54^ and Tophat^55^, respectively. After trimming low-quality cells (cells with <25,000 mapped reads or <1,000 genes), the remaining cells had a median of 122,000 mapped reads and 2,866 genes. Pre-processing and differential expression analysis were conducted in Seurat^44^ using the Wilcoxon rank-sum test. To test for differences in IFN responsiveness, participant-specific IFN response gene lists were used to generate scores in the pDCs using the AddModuleScore function in Seurat. The gene list used to score in each participant was chosen by including any gene that appeared at least twice in the modules that belonged to MM3 for that participant (see Extended Data Fig. 3i).

### Luminex and ELISA cytokine measurements

Matching plasma cytokine levels were determined in duplicate using a multiplexed magnetic bead assay (catalog no. LHC6003M, Life Technologies) in accordance with the manufacturer’s instructions. Briefly, a mixture of beads that were coated with anticytokine antibodies were prewashed and then incubated with the plasma samples. They were then co-incubated with a mixture of biotinylated detector antibodies followed by R-phycoerythrin (R-PE)-conjugated streptavidin. A magnetic separator was used to wash the beads between incubations. Fluorescence intensity was determined on a Bio-Plex 200 system. Concentrations of the cytokines in the samples were determined by interpolating on sigmoid four-parameter logistic regression standard curves.

Matching plasma soluble CD14 levels were measured using human CD14 DuoSet ELISA Kit (catalog no. DY383, R&D Systems) in accordance with the manufacturer’s instructions. Briefly, a 96-well microplate was coated with anti-CD14 capture antibody overnight. The plates were blocked with reagent diluent for 1 h and then incubated with recombinant standards or plasma samples (diluted 1:600 in reagent diluent) for 1.5 h. They were further incubated with detection antibody for 1.5 h, followed by streptavidin–HRP for 20 min. The substrate was added for 20 min for color development. The reaction was stopped by adding stop solution. Optical density (OD) of each well was determined at 450 nm (corresponding ODs at 530 nm were subtracted for wavelength correction). The concentrations of soluble CD14 in the samples were determined by interpolating on a sigmoid four-parameter logistic regression standard curve. All incubations were done at room temperature.

### T cell receptor CDR3 pulldown and analysis

To directly sequence the CDR3s from proliferating T cells assayed by Seq-Well, we applied a recently published TCR pulldown method^56^ to WTA products from the 2-week, 3-week and 4-week timepoint samples from all four participants. Briefly, biotinylated capture probes from the TRBC region were annealed to melted WTA cDNA. Magnetic streptavidin beads were then used to pull down cDNA enriched for TRBC; this cDNA was subsequently amplified using KAPA HiFi Mastermix (Kapa Biosystems) and purified using 0.75× SeraPure beads to select for 0.8–1-kb sized DNA fragments. To select for sequences with full CDR3 regions, a pool of V-region primers was used to further amplify sequences of interest. Step-out PCR was used to add sequencing handles and the resulting libraries were sequenced on a NextSeq 550 using a 150-cycle NextSeq kit with 148 cycles for Read 1 (CDR3) and 20 cycles for Index 1 (BC + UMI). Sequences of the primers used are available in Tu et al.^56^. CDR3 consensus sequences were aligned and determined as outlined previously. Across the entire dataset, we detected ~50% of TCR-β chain CDR3s.

### Determining the cellular identity of early proliferating cytotoxic cells

A recent scRNA-seq study on cytotoxic innateness characterized cytotoxic γδT and NK cells in healthy humans, noting basal levels of *TRDC* in both cell types^46^. To determine whether *TRDC*^*+*^*FCGR3A*^+^ cells were γδT or NK cells, we scored them, as well as nonproliferating CTLs and NK cells, against gene signatures described in that study (Extended Data Fig. 10g). Based on score similarity to NK cells, and the relative downregulation of CD3 compared to the other proliferating T cell subsets (FDR-corrected Wilcoxon rank-sum test; *CD3D:* log(FC) = −0.895, *q* = 2.7 × 10^−42^; *CD3G*: log(FC) = −0.923, *q* = 8.9 × 10^−37^), we determined cluster 4 (lilac) to be proliferating NK cells.

### Statistics

WGCNA module significance was tested using a permutation test (*n* = 10,000) on dissimilarity values and compared to the distribution of true values using a one-sided Mann–Whitney *U*-test. After FDR correction, modules that failed <500 tests (*P* < 0.05) were considered significant. Participant-specific modules were tested for temporal variation in score using a two-sided Mann–Whitney *U*-test with 1,000 subsamplings. Modules discovered across all participants were tested for temporal variation with binned samples using an ANOVA with two models: (1) null ~1 + participant; and (2) alternative ~1 + participant + time.bin (*P* < 0.05). Gene-set analysis was performed on GMs using IPA (Qiagen). For the differential expression analysis in monocytes, we performed hypergeometric tests using gene lists from MSigDB v.6.2 and corrected for multiple-hypothesis testing using the FDR procedure. Differential expression analysis between groups of single cells was performed using either a two-sided Wilcoxon rank-sum test or the ‘bimod’ test as implemented in Seurat.

### Reporting Summary

Further information on research design is available in the Nature Research Reporting Summary linked to this article.

## Online content

Any methods, additional references, Nature Research reporting summaries, source data, extended data, supplementary information, acknowledgements, peer review information; details of author contributions and competing interests; and statements of data and code availability are available at 10.1038/s41591-020-0799-2.

## Supplementary information

Supplementary Information Supplementary Tables 1, 2, and 11.

Reporting Summary

Supplementary Tables Supplementary Tables 3–10.

Supplementary Software R script with functions for module discovery and their application to a Seurat v.2.3.4 object.

## Data Availability

The expression matrix and associated metadata can be visualized and downloaded from the Alexandria Project, a Bill and Melinda Gates Foundation-funded portal (part of the Single-Cell Portal hosted by The Broad Institute of MIT and Harvard): https://singlecell.broadinstitute.org/single_cell/study/SCP256. It is also available for download, alongside accompanying supplementary material, at our website: http://shaleklab.com/resource/immune-dynamics-of-acute-hiv-infection. De-identified raw data are available on request through the corresponding authors, given the at-risk nature of people with HIV. The raw data will also be submitted to dbGaP, pending IRB approval.

## Code availability

The custom code used for GM discovery analysis is in the Supplementary Software.
