# Supplementary material for: Integrated single-cell analysis of multicellular immune dynamics during hyperacute HIV-1 infection
Source: Nat Med. 2020 Mar 23;26(4):511–8. doi: 10.1038/s41591-020-0799-2 (PMC7237067; doi:10.1038/s41591-020-0799-2)
Supplement: Supplementary file 1 — Supplementary Tables 1, 2, and 11. [file 41591_2020_799_MOESM1_ESM.pdf]

nature medicine

SUPPLEMENTARY INFORMATION

https://doi.org/10.1038/s41591-020-0799-2

In the format provided by the authors and unedited.

Integrated single-cell analysis of multicellular immune dynamics during hyperacute HIV-1 infection

Samuel W. Kazer[1,2,3,4], Toby P. Aicher[1,2,4], Daniel M. Muema[5,6], Shaina L. Carroll[7], Jose Ordovas-Montanes[1,2,3,4,8,9], Vincent N. Miao[1,2,4,10], Ang A. Tu[4,11,12], Carly G. K. Ziegler[1,2,4,10], Sarah K. Nyquist[1,2,4,13,14], Emily B. Wong[5,15,16,17], Nasreen Ismail[6], Mary Dong[1], Amber Moodley[6], Bonnie Berger[14,18], J. Christopher Love[1,11,12], Krista L. Dong[1], Alasdair Leslie[5,16], Zaza M. Ndhlovu[1,5,6,19], Thumbi Ndung'u[1,5,6,16,20], Bruce D. Walker[1,6,19,21] ✉ and Alex K. Shalek[1,2,3,4,10,11,13,21] ✉

[1]Ragon Institute of MGH, MIT and Harvard, Cambridge, MA, USA. [2]Institute for Medical Engineering and Science (IMES), Massachusetts Institute of Technology, Cambridge, MA, USA. [3]Department of Chemistry, Massachusetts Institute of Technology, Cambridge, MA, USA. [4]Broad Institute of MIT and Harvard, Cambridge, MA, USA. [5]African Health Research Institute, Durban, South Africa. [6]HIV Pathogenesis Programme, Nelson R. Mandela School of Medicine, Doris Duke Medical Research Institute, University of KwaZulu-Natal, Durban, South Africa. [7]Department of Molecular and Cell Biology, University of California, Berkeley, CA, USA. [8]Division of Gastroenterology, Boston Children's Hospital, Boston, MA, USA. [9]Harvard Stem Cell Institute, Cambridge, MA, USA. [10]Program in Health Sciences and Technology, Harvard Medical School & Massachusetts Institute of Technology, Boston, MA, USA. [11]Koch Institute for Integrative Cancer Research, Massachusetts Institute of Technology, Cambridge, MA, USA. [12]Department of Chemical Engineering, Massachusetts Institute of Technology, Cambridge, MA, USA. [13]Program in Computational and Systems Biology, Massachusetts Institute of Technology, Cambridge, MA, USA. [14]Computer Science and Artificial Intelligence Laboratory, Massachusetts Institute of Technology, Cambridge, MA, USA. [15]Division of Infectious Diseases, Massachusetts General Hospital, Boston, MA, USA. [16]Division of Infection and Immunity, University College London, London, UK. [17]Harvard Medical School, Boston, MA, USA. [18]Department of Mathematics, Massachusetts Institute of Technology, Cambridge, MA, USA. [19]Howard Hughes Medical Institute, Chevy Chase, MD, USA. [20]Max Planck Institute for Infection Biology, Berlin, Germany. [21]These authors contributed equally: Bruce D. Walker, Alex K. Shalek. ✉e-mail: BWALKER@mgh.harvard.edu; shalek@mit.edu

Supplementary Information for:

Integrated Single-Cell Analysis of Multicellular Immune Dynamics during Hyper-Acute HIV-1 Infection

Samuel W. Kazer[1,2,3,4], Toby P. Aicher[1,2,4], Daniel M. Muema[5,6], Shaina L. Carroll[7], Jose Ordovas-Montanes[1,2,3,4,8,9], Vincent N. Miao[1,2,4,10], Ang A. Tu[4,11,12], Carly G. K. Ziegler[1,2,4,10], Sarah K. Nyquist[1,2,4,13,14], Emily B. Wong[5,15,16], Nasreen Ismail[6], Mary Dong[1], Amber Moodley[6], Bonnie Berger[14,17], J. Christopher Love[1,11,12], Krista L. Dong[1], Alasdair Leslie[5,16], Zaza M. Ndhlovu[1,5,6,18], Thumbi Ndung'u[1,5,6,16,19], Bruce D. Walker[1,6,18,†,*], and Alex K. Shalek[1,2,3,4,10,11,13,†,*]

Affiliations

[1]Ragon Institute of MGH, MIT, and Harvard, Cambridge, MA, USA

[2]Institute for Medical Engineering and Science (IMES), Massachusetts Institute of Technology, Cambridge, MA, USA

[3]Department of Chemistry, Massachusetts Institute of Technology, Cambridge, MA, USA

[4]Broad Institute of MIT and Harvard, Cambridge, MA, USA

[5]African Health Research Institute, Durban, South Africa

[6]HIV Pathogenesis Programme, Nelson R. Mandela School of Medicine, Doris Duke Medical Research Institute, University of KwaZulu-Natal, Durban, South Africa

[7]Department of Molecular and Cell Biology, University of California, Berkeley, CA, USA

[8]Division of Gastroenterology, Boston Children's Hospital, Boston, MA, USA

[9]Harvard Stem Cell Institute, Cambridge, MA, USA

[10]Program in Health Sciences and Technology, Harvard Medical School and Massachusetts Institute of Technology, Boston, MA, USA

[11]Koch Institute for Integrative Cancer Research, Massachusetts Institute of Technology, Cambridge, MA, USA

[12]Department of Chemical Engineering, Massachusetts Institute of Technology, Cambridge, MA, USA

[13]Program in Computational and Systems Biology, Massachusetts Institute of Technology, Cambridge, MA, USA

[14]Computer Science and Artificial Intelligence Laboratory, Massachusetts Institute of Technology, Cambridge, MA, USA

[15]Division of Infectious Diseases, Massachusetts General Hospital, Boston, MA, USA; Harvard Medical School, Boston, MA, USA

[16]Division of Infection and Immunity, University College London, London, UK

[17]Department of Mathematics, Massachusetts Institute of Technology, Cambridge, MA, USA

[18]Howard Hughes Medical Institute, Chevy Chase, MD, USA

[19]Max Planck Institute for Infection Biology, Berlin, Germany

[†]These authors contributed equally to this work.

*Corresponding author emails: shalek@mit.edu (A.K.S.); BWALKER@mgh.harvard.edu (B.D.W.)

SUPPLEMENTARY TABLES

Individual	Time Point (days relative to first positive viral RNA test) *Pre-Infection*	*0 Weeks*	*1 Week*	*2 Weeks*	*3 Weeks*	*4 Weeks*	*6 Months*	*1 Year*
P1	-70	3	10	17	24	31	168	330
P2	-42	3	N.C.	17	24	31	161	329
P3	-35	1	7	14	21	28	162	329
P4	-95	1	7	14	24	N.C.	164	413

Individual	Age at Detection	STI at HIV Detection	Fiebig Stage at Detection	HLA-A	HLA-B	HLA-C
P1	24	Yes	I	24:02 / 29:02	07:02 / 44:03	07:01 / 07:02
P2	21	N/A	I	68:01 / 68:02	57:02 / 58:02	06:02 / 18
P3	24	Yes	I	02:05 / 66:01	14:01 / 39:10	8:04 / 12:03
P4	21	Yes	I	01:01 / 66:01	39:10 / 81:01	12:03 / 18

Supplementary Table 1: Time point, clinical information, and HLA genotype for participants studied.

P1	Pre-Infection	0 Weeks	1 Week	2 Weeks	3 Weeks	4 Weeks	6 Months	1 Year
B cells	133	249	22	194	134	315	242	241
CD4+ T cells	899	606	157	389	294	669	718	751
CTLs	144	138	50	277	247	487	404	396
DCs	72	14	2	19	23	42	37	79
Monocytes	547	622	162	571	450	692	409	876
NK cells	175	143	92	422	308	422	341	512
Plasmablasts	6	9	4	23	4	16	9	0
**Total Cells**	1976	1781	489	1895	1460	2643	2160	2855

P2	Pre-Infection	0 Weeks	1 Week	2 Weeks	3 Weeks	4 Weeks	6 Months	1 Year
B cells	171	58	N/A	23	169	54	109	157
CD4+ T cells	869	231	N/A	63	37	144	83	567
CTLs	170	120	N/A	178	249	103	160	259
DCs	93	121	N/A	20	44	48	85	42
Monocytes	1324	1745	N/A	587	691	538	952	1664
NK cells	85	64	N/A	136	215	70	79	152
Plasmablasts	8	11	N/A	268	41	29	31	11
**Total Cells**	2720	2350	0	1275	1446	986	1499	2852

P3	Pre-Infection	0 Weeks	1 Week	2 Weeks	3 Weeks	4 Weeks	6 Months	1 Year
B cells	154	95	53	88	138	138	53	76
CD4+ T cells	967	245	272	305	612	501	160	341
CTLs	278	236	311	520	608	474	100	131
DCs	29	5	37	34	47	29	5	13
Monocytes	920	1017	1122	1214	577	1361	146	893
NK cells	151	166	270	343	119	171	70	62
Plasmablasts	57	18	41	72	37	30	7	8
**Total Cells**	2556	1782	2106	2576	2138	2704	541	1524

P4	Pre-Infection	0 Weeks	1 Week	2 Weeks	3 Weeks	4 Weeks	6 Months	1 Year
B cells	60	61	22	42	58	N/A	174	166
CD4+ T cells	100	155	124	391	87	N/A	232	1041
CTLs	58	71	92	537	267	N/A	120	155
DCs	94	93	33	29	70	N/A	30	50
Monocytes	1277	1788	2014	618	2286	N/A	1199	869
NK cells	18	35	59	214	24	N/A	61	88
Plasmablasts	274	9	43	97	27	N/A	9	4
**Total Cells**	1881	2212	2387	1928	2819	0	1825	2373

Supplementary Table 2: Cell frequencies by time point and participant.

B	Plasmablast	CD4	NK	Monocyte	CD1C+ DC	CTL	Prolif. T
LYZ	*LYZ*	*LYZ*	*LYZ*	*CD3E*	*CD3E*	*LYZ*	*LYZ*
CD14	*CD14*	*CD14*	*CD14*	*CD3G*	*CD3G*	*CD14*	*CD14*
TRBC2	*TRBC2*	*IGJ*	*IGJ*	*CD3D*	*CD3D*	*IGJ*	*IGJ*
TRDC	*TRDC*	*IGHM*	*IGHM*	*IGJ*	*IGJ*	*IGHM*	*IGHM*
CD3E	*CD3E*	*IGHG1*	*IGHG1*	*IGHM*	*IGHM*	*IGHG1*	*IGHG1*
CD3G	*CD3G*	*IGHG2*	*IGHG2*	*IGHG1*	*IGHG1*	*IGHG2*	*IGHG2*
CD3D	*CD3D*	*IGHG3*	*IGHG3*	*IGHG2*	*IGHG2*	*IGHG3*	*IGHG3*
CCL5	*CCL5*	*IGHG4*	*IGHG4*	*IGHG3*	*IGHG3*	*IGHG4*	*IGHG4*
MNDA		*IGHD*	*IGHD*	*IGHG4*	*IGHG4*	*IGHD*	*IGHD*
VCAN		*IGHA1*	*IGHA1*	*IGHD*	*IGHD*	*IGHA1*	*IGHA1*
FCGR3A		*FCGR3A*	*CD3E*	*IGHA1*	*IGHA1*		
NKG7		*VCAN*	*CD3D*	*TRBC2*	*TRBC2*		
			CD3G	*TRDC*	*TRDC*		
			TRAC				

Supplementary Table 11: Genes used to determine residual RNA contamination in each cell type.
