# Supplementary material for: Integrated single-cell analysis of multicellular immune dynamics during hyperacute HIV-1 infection
Source: Nat Med. 2020 Mar 23;26(4):511–8. doi: 10.1038/s41591-020-0799-2 (PMC7237067; doi:10.1038/s41591-020-0799-2)
Supplement: Supplementary file 2 — Reporting Summary [file 41591_2020_799_MOESM2_ESM.pdf]

nature research

Corresponding author(s): Alex K. Shalek, Bruce D. Walker

Last updated by author(s): Jan 21, 2020

Reporting Summary

Nature Research wishes to improve the reproducibility of the work that we publish. This form provides structure for consistency and transparency in reporting. For further information on Nature Research policies, see Authors & Referees and the Editorial Policy Checklist.

Statistics

For all statistical analyses, confirm that the following items are present in the figure legend, table legend, main text, or Methods section.

n/a	Confirmed	
☐	☒	The exact sample size (n) for each experimental group/condition, given as a discrete number and unit of measurement
☐	☒	A statement on whether measurements were taken from distinct samples or whether the same sample was measured repeatedly
☐	☒	The statistical test(s) used AND whether they are one- or two-sided<br>*Only common tests should be described solely by name; describe more complex techniques in the Methods section.*
☐	☒	A description of all covariates tested
☐	☒	A description of any assumptions or corrections, such as tests of normality and adjustment for multiple comparisons
☐	☒	A full description of the statistical parameters including central tendency (e.g. means) or other basic estimates (e.g. regression coefficient) AND variation (e.g. standard deviation) or associated estimates of uncertainty (e.g. confidence intervals)
☐	☒	For null hypothesis testing, the test statistic (e.g. F, t, r) with confidence intervals, effect sizes, degrees of freedom and P value noted<br>*Give P values as exact values whenever suitable.*
☒	☐	For Bayesian analysis, information on the choice of priors and Markov chain Monte Carlo settings
☒	☐	For hierarchical and complex designs, identification of the appropriate level for tests and full reporting of outcomes
☐	☒	Estimates of effect sizes (e.g. Cohen's d, Pearson's r), indicating how they were calculated

Our web collection on statistics for biologists contains articles on many of the points above.

Software and code

Policy information about availability of computer code

Data collection	Sequencing data was collected using a NextSeq500. Flow cytometry data was collected on a BD SORP FACSAria II cell sorter using BD FACSDiva software (version 8.0.2).
Data analysis	Reads were aligned against hg19 (Ensembl: Homo_sapiens.GRch37.74) using Drop-seq Tools (v.1.13). After alignment, data were processed using R (v3.4.4), Seurat (v.2.3.4), WGCNA (v1.66), Mfuzz (v.2.38.0), and custom scripts provided in the Supplemental Software. Gene Set Analysis was performed using Ingenuity Pathway Analysis (Qiagen, Summer 2019 Release) and MSigDB (v6.2). Flow cytometry data was analyzed using Flowjo (v10.4).

For manuscripts utilizing custom algorithms or software that are central to the research but not yet described in published literature, software must be made available to editors/reviewers. We strongly encourage code deposition in a community repository (e.g. GitHub). See the Nature Research guidelines for submitting code & software for further information.

Data

Policy information about availability of data

All manuscripts must include a data availability statement. This statement should provide the following information, where applicable:

- Accession codes, unique identifiers, or web links for publicly available datasets
- A list of figures that have associated raw data
- A description of any restrictions on data availability

The expression matrix and associated meta data can be visualized and downloaded from The Alexandria Project, a Bill and Melinda Gates Foundation funded portal (part of the Single Cell Portal hosted by The Broad Institute of MIT and Harvard): https://singlecell.broadinstitute.org/single_cell/study/SCP256. De-identified raw data is available upon request through the corresponding authors given the at-risk nature of HIV infected persons. The raw data will also be submitted to dbGaP pending IRB approval.

Field-specific reporting

Please select the one below that is the best fit for your research. If you are not sure, read the appropriate sections before making your selection.

☒ Life sciences ☐ Behavioural & social sciences ☐ Ecological, evolutionary & environmental sciences

For a reference copy of the document with all sections, see nature.com/documents/nr-reporting-summary-flat.pdf

Life sciences study design

All studies must disclose on these points even when the disclosure is negative.

Sample size	Sample size was selected based on limited sample availability from the FRESH study (n=4). We chose participants for which we had at least 7/8 of the following time points with samples remaining: pre-infection, HIV detection (0 weeks), 1 week, 2 weeks, 3 weeks, 4 weeks, 6 months, and 1 year post HIV detection. With longitudinal sampling, we have 30 independent samples. Within an individual, we have 7-8 samples, which provided robust statistics based on cell number replicates (~14k-16k cells per participant).
Data exclusions	Cells with fewer than 750 or more than 6,000 unique transcript reads were removed from analysis as low quality cells or potential doublets; these cells would add unwanted noise to downstream analysis. Any cells whose measured transcriptome was greater than 20% mapping to mitochondrial genes were also removed, as these have been shown to be low quality cells. Both exclusion criteria were pre-established for this data analysis.
Replication	Every sample was processed by Seq-Well in duplicate (except for one: P1 - 6 months, due to experiment failure). In the majority of cases, cell frequencies were confirmed by duplicate measurements. Due to sample limitations and current standard of care for HIV infection, we were unable to assay additional individuals to further validate our findings above the participants described here (n=4).
Randomization	All participants contracted HIV-infection in this study. Pre-infection samples were determined by negative RT-PCR of viral RNA and other HIV tests. All samples classified as post-HIV detection were characterized by positive RT-PCR of viral HIV RNA in the plasma. Controller phenotype was determined by a HIV viral load of fewer than 1,000 viral copies/mL for 3 consecutive tests, spaced 6 months apart, starting at 1.8 years post initial infection.
Blinding	Blinding was not relevant as there was no placebo group. All HIV- samples were collected prior to infection as participant-internal controls.

Reporting for specific materials, systems and methods

We require information from authors about some types of materials, experimental systems and methods used in many studies. Here, indicate whether each material, system or method listed is relevant to your study. If you are not sure if a list item applies to your research, read the appropriate section before selecting a response.

Materials & experimental systems

n/a	Involved in the study
☐	☒ Antibodies
☒	☐ Eukaryotic cell lines
☒	☐ Palaeontology
☒	☐ Animals and other organisms
☐	☒ Human research participants
☒	☐ Clinical data

Methods

n/a	Involved in the study
☒	☐ ChIP-seq
☐	☒ Flow cytometry
☒	☐ MRI-based neuroimaging

Antibodies

Antibodies used	Alexa Fluor 700 - CD45; Biolegend; clone 2D1; Cat# 368514; Lot B 248834; 1:200 Dilution<br>BUV737 – CD3; BD Biosciences; clone UCHT1; Cat# 564307; Lot 7335654; 1:200 Dilution<br>BV711 – CD4; Biolegend; clone OKT4; Cat# 317440; Lot B249480; 1:200 Dilution<br>BUV395 – CD8; BD Biosciences; clone RPA-T8; Cat# 563795; Lot 7069910; 1:200 Dilution<br>BV605 – CD14; Biolegend; clone M5E2; Cat# 301834; Lot B247581; 1:200 Dilution<br>BV510 – HLA-DR; BD Biosciences; clone G46-6; Cat# 563083; Lot B249262; 1:200 Dilution<br>BV650 – CD123; Biolegend; clone 6H6; Cat# 306020; Lot B241226; 1:200 Dilution<br>APC-Cy7 – CD11c; Biolgened; clone 2D1; Cat# 337218; Lot B254813; 1:200 Dilution
Validation	Alexa Flour 700 – CD45. Validated by Biolegend on human peripheral blood lymphocytes (mouse IgG1 K control).<br>BUV737 – CD3. Validated by BD Biosciences on human peripheral blood lymphocytes (mouse IgG1 K control).<br>BV711 – CD4. Validated by Biolegend on human peripheral blood lymphocytes (mouse IgG1 K control).<br>BUV395 – CD8. Validated by BD Biosciences on human peripheral blood lymphocytes (mouse IgG1 K control).<br>BV605 – CD14. Validated by Biolegend on human peripheral blood lymphocytes (mouse IgG1 K control).<br>BV510 – HLA-DR. Validated by BD Biosciences on human peripheral blood lymphocytes and monocytes (mouse IgG1 K control).

BV650 – CD123. Validated by Biolegend on human peripheral blood leukocytes (mouse IgG1 K control).
APC-Cy7 – CD11c. Validated by Biolegend on human peripheral blood granulocytes (mouse IgG1 K control).
All validation information is available on the Biolegend and BD Biosciences websites:
https://www.biolegend.com/
https://www.bdbiosciences.com/en-us

Human research participants

Policy information about studies involving human research participants

Population characteristics	For P1, P2, P3, P4 respectively:<br>Age at HIV Detection: 24, 21, 24, 21.<br>Gender: F, F, F, F.<br>Feibig Stage at HIV Detection: I, I, I, I.<br>HIV Status at enrollment: HIV-, HIV-, HIV-, HIV-.<br>Controller Phenotype: No, No, Yes, Yes.<br>HLA-A: 24:02/29:02, 68:01/68:02, 02:05/66:01, 01:01/66:01.<br>HLA-B: 07:02/44:03, 57:02/58:02, 14:01/39:10, 39:10/81:01.<br>HLA-C: 07:01/07:02, 06:02/18, 8:04/12:03, 12:03/18.
Recruitment	Eligible women were HIV uninfected, aged 18–23 years, sexually active, not pregnant, non-anemic (hemoglobin ≥10 g/L), without other barriers to participation (serious chronic illness, enrollment in another study, or family responsibilities), and gave written consent to enrollment. We targeted disadvantaged, at-risk women, favoring the enrollment of those who were unemployed and not attending school. Participants were recruited at local sites frequented by young people, including cafes, nightclubs, and shopping malls. (see Dong et al., The Lancet, 2018). Selection bias in participant recruitment is possible given the monetary incentive to join the study. This may have led to increased numbers of women seeking socio-economic gain. Nevertheless, in our study of n=4, we cannot comment on how any biases may impact the results herein, especially given our pre-infection control samples.
Ethics oversight	Biomedical research ethics committee of the University of KwaZulu-Natal and the IRB of Massachusetts General Hospital.

Note that full information on the approval of the study protocol must also be provided in the manuscript.

Flow Cytometry

Plots

Confirm that:

☒ The axis labels state the marker and fluorochrome used (e.g. CD4-FITC).

☒ The axis scales are clearly visible. Include numbers along axes only for bottom left plot of group (a 'group' is an analysis of identical markers).

☒ All plots are contour plots with outliers or pseudocolor plots.

☒ A numerical value for number of cells or percentage (with statistics) is provided.

Methodology

Sample preparation	PBMCs were purified from whole blood by ficoll phase separation, and subsequently frozen for long term LN2 storage. Frozen peripheral blood mononuclear cells (PBMCs) were thawed and washed twice with warm RPMI supplemented with 10% fetal bovine serum. Next, the cells were resuspended in FACS buffer (PBS supplemented with 1% FBS) and stained with antibodies on ice for 30 minutes in FACS buffer. Afterward, the cells were washed and stained with the viability stain Calcein Blue, AM (Invitrogen, C34853) for 15 minutes on ice. Finally, the stained cells were washed twice with FACS buffer.
Instrument	BD SORP FACSAria II
Software	BD FACSDiva (v8.0.2) was used to collect flow cytometry data. Subsequent analysis was performed using FlowJo v10.4.
Cell population abundance	Up to 250,000 viable immune cells (CD45+Calcein Blue+) were sorted into 1 ml of RPMI + 10% FBS for Seq-Well. For For Smart-Seq2 of pDCs, cells were directly sorted into 10 µl of RLT (Qiagen) + 1% BME in 96 well plates (24-96 per sample).
Gating strategy	FSC-A and SSC-A were gated to liberally include all lymphocytes and myeloid cells. Subsequent gating on intact cells was performed on FSC-W, and on singlets by SSC-W.<br>Live, Immune cells: Calcein+CD45+<br>CD4+ T cells: Calcein+CD45+CD14–CD3+CD4+.<br>CD8+ T cells: Calcein+CD45+CD14–CD3+CD8+.<br>pDCs: Calcein+CD45+CD14–CD3–CD11c–HLA-DR+CD123++.

☒ Tick this box to confirm that a figure exemplifying the gating strategy is provided in the Supplementary Information.
